# *Trans*-cyclosulfamidate mannose-configured cyclitol allows isoform-dependent inhibition of GH47 α-d-mannosidases through a bump–hole strategy†

**DOI:** 10.1039/d3sc05016e

**Published:** 2023-11-17

**Authors:** Alexandra Males, Ken Kok, Alba Nin-Hill, Nicky de Koster, Sija van den Beukel, Thomas J. M. Beenakker, Gijsbert A. van der Marel, Jeroen D. C. Codée, Johannes M. F. G. Aerts, Herman S. Overkleeft, Carme Rovira, Gideon J. Davies, Marta Artola

**Affiliations:** a York Structural Biology Laboratory, Department of Chemistry, The University of York York YO10 5DD UK gideon.davies@york.ac.uk; b Department of Medical Biochemistry, Leiden Institute of Chemistry (LIC), Leiden University P. O. Box 9502 2300 RA Leiden The Netherlands m.e.artola@lic.leidenuniv.nl; c Departament de Química Inorgànica i Orgànica (Secció de Química Orgànica), Institut de Química Teòrica i Computacional (IQTCUB), Universitat de Barcelona Martí i Franquès 1 08028 Barcelona Spain c.rovira@ub.edu; d Fundació Catalana de Recerca i Estudis Avançats (ICREA) Passeig Lluís Companys 23 08010 Barcelona Spain; e Department of Bio-organic Synthesis, Leiden Institute of Chemistry (LIC), Leiden University Einsteinweg 55 2333 CC Leiden The Netherlands

## Abstract

Class I inverting exo-acting α-1,2-mannosidases (CAZY family GH47) display an unusual catalytic itinerary featuring ring-flipped mannosides, ^3^*S*_1_ → ^3^*H*_4_^‡^ → ^1^*C*_4_. Conformationally locked ^1^*C*_4_ compounds, such as kifunensine, display nanomolar inhibition but large multigene GH47 mannosidase families render specific “isoform-dependent” inhibition impossible. Here we develop a bump-and-hole strategy in which a new mannose-configured 1,6-*trans*-cyclic sulfamidate inhibits α-d-mannosidases by virtue of its ^1^*C*_4_ conformation. This compound does not inhibit the wild-type GH47 model enzyme by virtue of a steric clash, a “bump”, in the active site. An L310S (a conserved residue amongst human GH47 enzymes) mutant of the model *Caulobacter* GH47 awoke 574 nM inhibition of the previously dormant inhibitor, confirmed by structural analysis of a 0.97 Å structure. Considering that L310 is a conserved residue amongst human GH47 enzymes, this work provides a unique framework for future biotechnological studies on *N*-glycan maturation and ER associated degradation by isoform-specific GH47 α-d-mannosidase inhibition through a bump-and-hole approach.

## Abstract 2

1,6-*Trans*-manno-cyclosulfamidate 6 inhibits selectively an L310S mutant of *Caulobacter* GH47 α-d-mannosidase by virtue of its ^1^*C*_4_ conformation and bump-and-hole strategy, enabling allele-specific inhibition within the GH47 α-mannosidase family.

## Graphical abstract

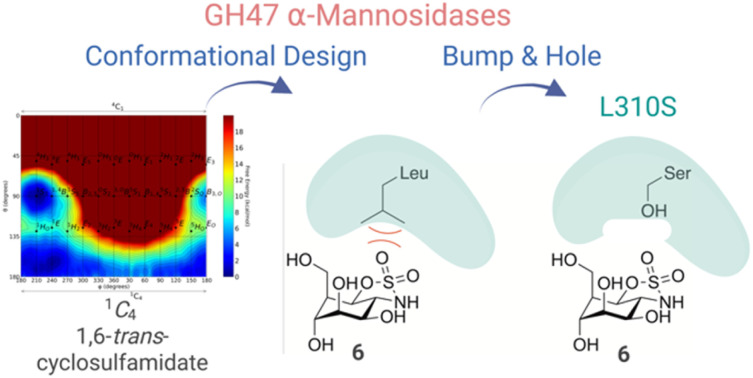

## Introduction

Selective small molecule inhibition of glycoside hydrolases (“glycosidases”) has proved to be an essential tool to unlock their cellular functions. Classical inhibitor design approaches involve transition-state mimicry through charge and conformation approaches,^1–4^ targeting the (catalytic) nucleophile in specific mechanisms^2,5,6^ or unusual reaction mechanisms (such as the neighbouring group participation of the *O*-GlcNAc hydrolase^7^). All these approaches aim to enable specific inhibition of target carbohydrate-active enzymes, which consist of 1–2% of any genome. Glycosidases are a diverse class of enzymes, with sequence-based approaches currently classifying over 160 distinct families in the CAZy database.^8^ This classification allows for a family-wide definition of enzyme mechanisms and may predict general conformational itineraries.^8,9^ Initially described by Koshland in 1953,^10^ and with few exceptions, glycosidases follow two distinct reaction pathways leading to inversion or net retention of the configuration of the anomeric carbon after hydrolysis. Retaining enzymes (typically) harness a double-displacement mechanism *via* the formation and subsequent breakdown of a covalent intermediate flanked by an oxocarbenium-ion-like transition state, a mechanism which can be specifically hijacked using covalent inactivators.^2,11,12^ In contrast, the one-step inverting mechanism involves an “S_N_2” direct attack of water at the anomeric carbon through a single oxocarbenium ion transition-state rendering small molecule selective inhibition more challenging.^13^ Regardless of the reaction mechanism, one serious challenge, addressed here for the inverting α-d-mannosidases, is the difficulty in specifically inhibiting single members of closely related enzyme families, whose active centres are identical, and for whom all members of the superfamily are inhibited similarly by small molecules.

**Fig. 1:**
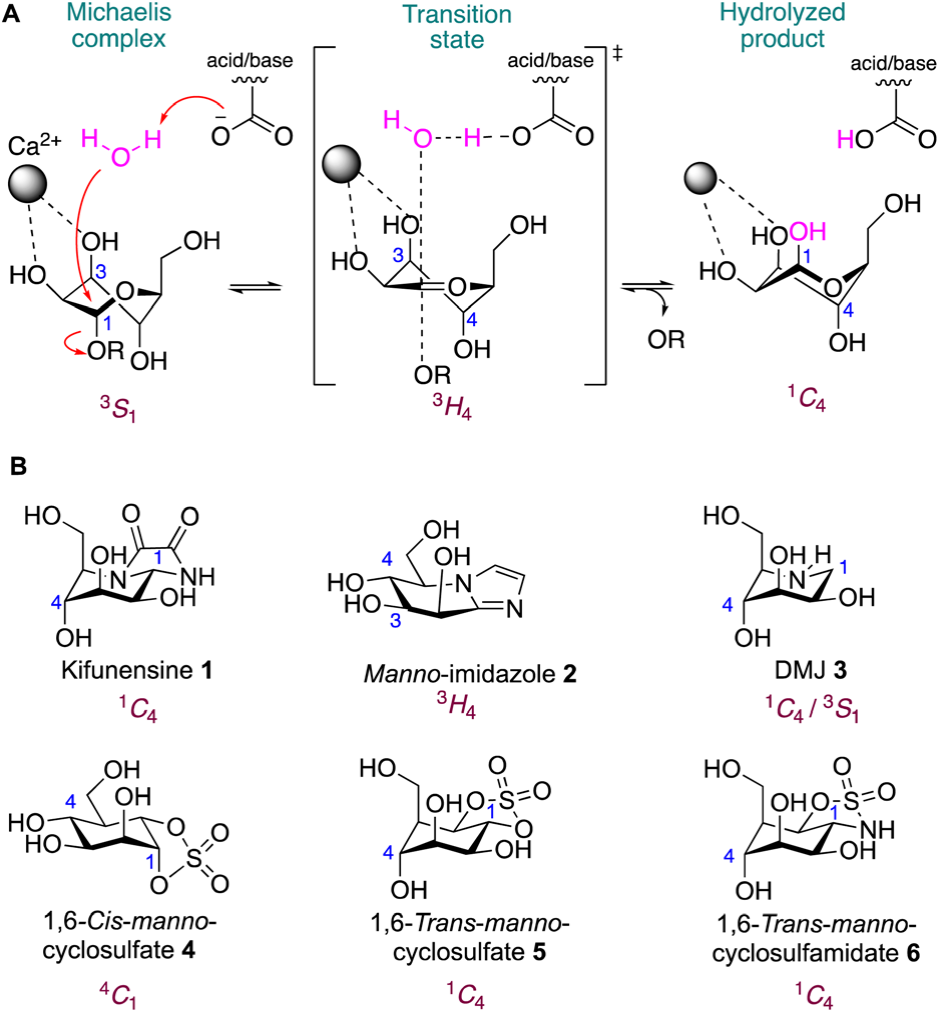
(A) Conformational reaction itinerary of inverting GH47 α-d-mannosidases. (B) Chemical structures of known inhibitors kifunensine 1, mannoimidazole 2, DMJ 3 and new molecules developed in this work 1,6-*cis*-manno-cyclosulfate 4, 1,6-*trans*-manno-cyclosulfate 5 and 1,6-*trans*-manno-cyclosulfamidate 6.

Decades of work on diverse glycosidases (including α-d-mannosidases,^14–16^ reviewed by Williams and collaborators^3,17^) has shown that the substrates undergo specific conformational fluctuations to accommodate their steric and electronic features. Indeed, conformational mimicry of the ligands along the reaction coordinates, Michaelis complex, transition state or product complexes, has been demonstrated to be of key relevance when designing inhibitors for specific carbohydrate hydrolases. GH47 mannosidases are Ca^2+^-dependent metalloenzymes that follow a one-step inverting mechanism with a ^3^*S*_1_ (Michaelis complex) → ^3^*H*_4_^‡^ (transition state) → ^1^*C*_4_ (product) conformational itinerary (Fig. 1A).^18,19^ Kifunensine 1 is one such conformationally restrained compound that achieves selective inhibition of the GH47 α-d-mannosidase family by virtue of mimicry of the mannose configuration and ^1^*C*_4_ conformation of the enzyme-product state (Fig. 1).^15^ Similarly, mannoimidazole 2 and 1-deoxymannojirimycin (DMJ) 3 also inhibit GH47 α-d-mannosidases, but do so by mimicking the oxocarbenium transition state (^3^*H*_4_^‡^) or both the Michaelis complex (^3^*S*_1_) and product (^1^*C*_4_) conformations, respectively (Fig. 1). However, all are incapable of inhibiting individual GH47 α-d-mannosidases selectively which means they cannot be used for specific inhibition of single enzymes in cells.

**Fig. 2:**
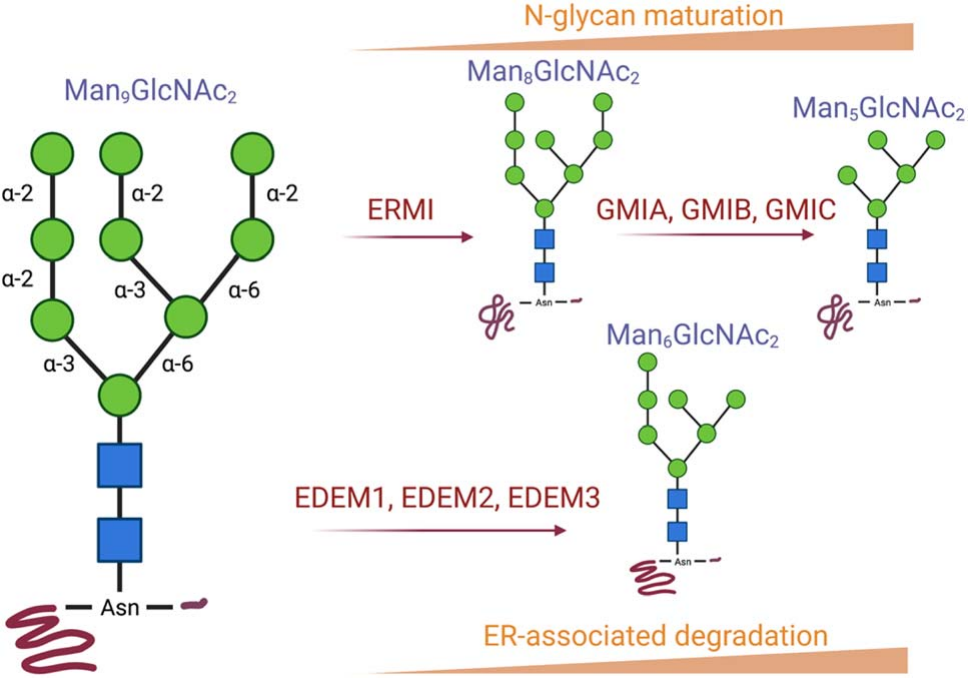
GH47 Class I inverting α-d-mannosidase is composed of seven enzymes: α-1,2-mannosidases ERMI, GMIA, GMIB, and GMIC are involved in *N*-glycan maturation whereas EDEM1, EDEM2 and EDEM3 play a key role in the ER-associated degradation (ERAD) machinery.

GH47 Class I inverting α-d-mannosidases hydrolyse 1,2-mannosidic linkages and are responsible for the processing of *N*-glycans, ultimately regulating the maturation and quality control of glycoproteins in the secretory pathway.^20^ GH47 mannosidases can be divided into three subfamilies within the endoplasmic reticulum (ER) and Golgi apparatus. The cleavage of α-1,2-mannoside linkages in Man9GlcNAc2-Asn substrates is initiated by ERMI (first subfamily) followed by Golgi-α-1,2-mannosidases GMIA, GMIB, and GMIC (second subfamily), hydrolysing subsequent α-1,2-mannoside branches and affording Man5GlcNAc2-Asn (Fig. 2). The third subfamily comprises of ER degradation enhancing α-mannosidase-like (EDEM) enzymes (EDEM1, EDEM2 and EDEM3) that target misfolded proteins and mark them for degradation *via* the ER-associated degradation (ERAD) machinery.

Given the success of generic conformational mimicry for enzymes of this family^15,19,21^ (notwithstanding the fact that humans have a large multigene family of seven GH47 enzymes) we sought to test if cyclic sulfates and sulfamidates might possess similar conformational attributes. Recently, we demonstrated that a cyclophellitol analogue bearing a *cis*-cyclic sulfate electrophile is a selective nanomolar covalent α-glucosidase inhibitor by virtue of its ^4^*C*_1_ Michaelis complex mimicry.^2^ Substitution of this cyclic sulfate by an unreactive 1,6-cyclosulfamidate yielded diverse α-glucosidase^22^ and α-galactosidase^23^ competitive inhibitors with great potential as enzyme stabilizers for the treatment of lysosomal storage disorders such as Pompe or Fabry disease. In this work, we sought to build upon the capability of *cis*-cyclic sulfates and sulfamidates to lock their compounds in a ^4^*C*_1_ chair conformation^2,22–24^ and hypothesize that *trans*-cyclic sulfate 5 and sulfamidate 6 may instead invert this chair, yielding a new class of ^1^*C*_4_ locked conformational glycosidase inhibitors (Fig. 1B).

Here, we present the design and synthesis of an α-d-mannose configured 1,6-*trans*-cyclic sulfamidate as a potential inhibitor of the GH47 α-d-mannosidase family. We show, using QM calculations of free energy landscapes, that cyclosulfamidate 6 favours a ^1^*C*_4_ chair conformation consistent with the GH47 conformational itinerary. We demonstrate that a steric clash in the enzyme active centre enables the implementation of a bump-and-hole methodology for GH47 inhibition. Mutation of a leucine, conserved across the family, to serine unlocks the dormant inhibition of 6 realising a 574 nM inhibitor specific for the mutant enzyme only. The work, combining conformational mimicry and bump-and-hole yields the opportunity for specific inhibition of individual, but closely related, α-d-mannosidases within the GH47 family. This proof of concept offers a singular system for future biotechnological investigations into *N*-glycan maturation and ER-associated degradation in diverse species.

## Results and discussion

**Fig. 3:**
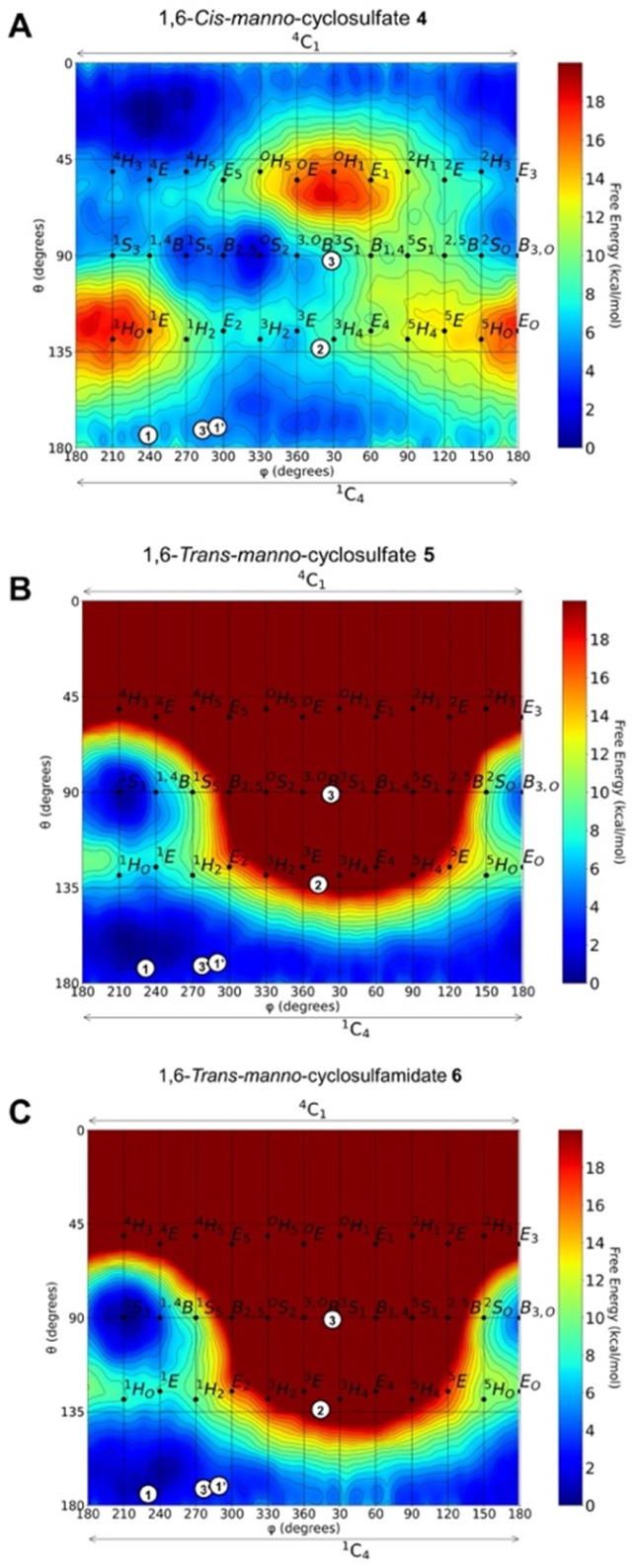
Conformational free energy landscapes (FELs, Mercator projection) contoured at 1 kcal mol^−1^ of (A) 1,6-*cis*-manno-cyclosulfate 4, (B) 1,6-*trans*-manno-cyclosulfate 5 and (C) 1,6-*trans*-manno-cyclosulfamidate 6. Symbols plot the observed conformations of known GH47 inhibitors in their respective enzyme complexes. Kifunensine bound to human ER I or *Ck*GH47 α-mannosidases (1, PDB 1FO3 or 1′, PDB 5NE5). Manno-imidazole bound to *Caulobacter* strain K31 α-mannosidase (*Ck*GH47) (2, PDB 4AYQ). DMJ bound to *Ck*GH47 (3 & 3′, PDB 5MEH). Further information on the most stable conformers and the corresponding Stoddart representations are provided in Fig. S1–S3.†

We first analysed, *in silico*, the intrinsic conformational preference of mannose-configured cyclic sulfates and sulfamidate 4–6, for which we employed QM metadynamics simulations to reconstruct a free energy landscape (FEL). The FELs show that opposite to the ^4^*C*_1_ conformation adopted by 1,6-*cis*-cyclic sulfate 4 (Fig. 3A), both *trans*-5 and 6 have a strong conformational preference for ^1^*C*_4_ (Fig. 3B and C). The sugar ring of 4 is quite flexible, but the relaxed chair conformation (^4^*C*_1_) is the most stable, followed by *B*_2,5_ and ^1^*C*_4_, which are ≈3 kcal mol^−1^ higher in energy. On the contrary, the FELs of 5 and 6 show that the 1,6-*trans* compounds are highly confined in the southern hemisphere. Both ^1^*S*_3_ and ^1^*C*_4_ conformations are thermally accessible, but ^1^*C*_4_ is the most stable. Given the preference for this conformation, we next sought to synthesize and establish whether, similar to kifunensine, the manno-configured 1,6-*trans*-cyclic sulfate 5 and/or sulfamidate 6 would act as a GH47 α-d-mannosidase inhibitor.

**Scheme 1:**
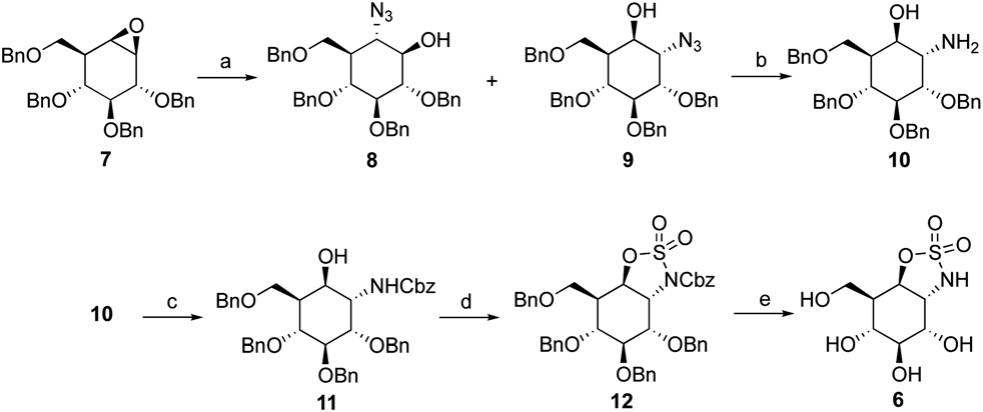
Synthesis of 1,6-*trans*-manno-cyclosulfamidate 6. Reagents and conditions: (a) NaN_3_, LiClO_4_, DMF, 100 °C, 14% (8) and 58% (9); (b) PtO_2_, H_2_, THF, rt, 86%; (c) benzyl chloroformate, K_2_CO_3_, dioxane, H_2_O, rt, 74%; (c) (i) SOCl_2_, Et_3_N, imidazole, DCM, 0 °C; (ii) RuCl_3_·H_2_O, NaIO_4_, ACN, EtOAc, H_2_O, 46%; (d) Pd/C, H_2_, MeOH, 75%.

1,6-*Cis*-manno-cyclosulfate, 4, and 1,6-*trans*-manno-cyclosulfate, 5, were synthesized from mannose-configured *cis*-diol and *trans*-diol, respectively (ESI, Scheme S1†). Though the final compounds 4 and 5 could be characterized, their storage for a week at room temperature led to compound degradation probably by intramolecular attack of the 2-OH pseudoanomeric position and opening of the cyclic sulfates. The synthesis of the potentially more stable cyclosulfamidate 6 started with the addition of sodium azide to tetra-*O*-benzyl-cyclophellitol 7 which resulted in a mixture of diastereomers 8 and 9. Azide 9 was subsequently reduced using PtO_2_ to obtain amine 10. Cbz-protection of the amine gave intermediate 11 which was treated with SOCl_2_ followed by RuCl_3_/NaIO_4_-mediated oxidation of the formed sulfites, resulting in the formation of fully protected *trans*-sulfamidate 12. *Trans*-sulfamidate 12 was then exposed to hydrogenation conditions to obtain cyclosulfamidate 6 which proved to be chemically stable (Scheme 1).

To our surprise, no significant inhibition of the GH47 model enzyme (whose active centre is identical to the human enzymes) from *Caulobacter* K31 strain was observed even at high inhibitor concentrations; 82% of α-1,2-mannobiose was hydrolysed by the WT GH47 mannosidase to mannose after the addition of 1 mM 6 (Fig. S4†) (in contrast to kifunensine (*K*_D_ of 39 nM),^15^ mannoimidazole (*K*_D_ of 47 nM),^19^ and DMJ (*K*_D_ of 481 nM)^21^). Accordingly, crystal soaks revealed no binding. Simple overlay of 6 over published complexes of the *Caulobacter* GH47 enzyme revealed a likely steric clash between one of the oxygen atoms of the sulfamidate group of 6 with the Cδ2 atom of the leucine side chain L310 in the active centre of the enzyme (Fig. S5†).

Although this initial observation was fortuitous, it immediately presented a solution to the problem of specific inhibition of individual enzymes. In contrast to other known GH47 inhibitors, we could exploit the clash to formulate a “bump-and-hole” strategy allowing selective individual α-mannosidase GH47 inhibition, otherwise not possible through chemical knockdown. Originally developed for kinases by Shokat,^25,26^ bump-and-hole engineering is best applied to large multigene families where isoform-specific inhibition (sometimes described as “allele specific”, reflecting the inhibition of one of a panel of closely related proteins encoded by closely related genes) is challenging. This strategy relies on introducing a “bump” on the inhibitor/ligand/substrate that is accommodated specifically by enzyme variants into which a complementary “hole” has been created through mutagenesis. Within the context of glycoscience, while Karanicolas's team expanded the approach to incorporate new allosteric pockets into the catalytic sites of several glycosidases,^27,28^ Hou *et al.* capitalized on the bump and hole strategy to precisely deliver nitric oxide using alkylated β-galacatosyl NONOates^29^ and Schumann *et al.* incorporated chemically tagged sugars into the cell surface glycome of living cells using “holed” glycosyltransferases.^30,31^ Since, despite conformational matching, 6 failed because of encountering a “bump” with L310, we hypothesized that exchanging the leucine for a smaller amino-acid would form an appropriate “hole” to accommodate 6 in a variant-specific manner. Importantly, L310 is an entirely conserved residue across all seven human GH47 enzymes (Fig. S7 and S8†). To demonstrate proof-of-concept, mutation to a serine was selected because of the potential to form a hydrogen bond to the inhibitor in addition to removing the steric clashing distance: a hole with benefits.

**Fig. 4:**
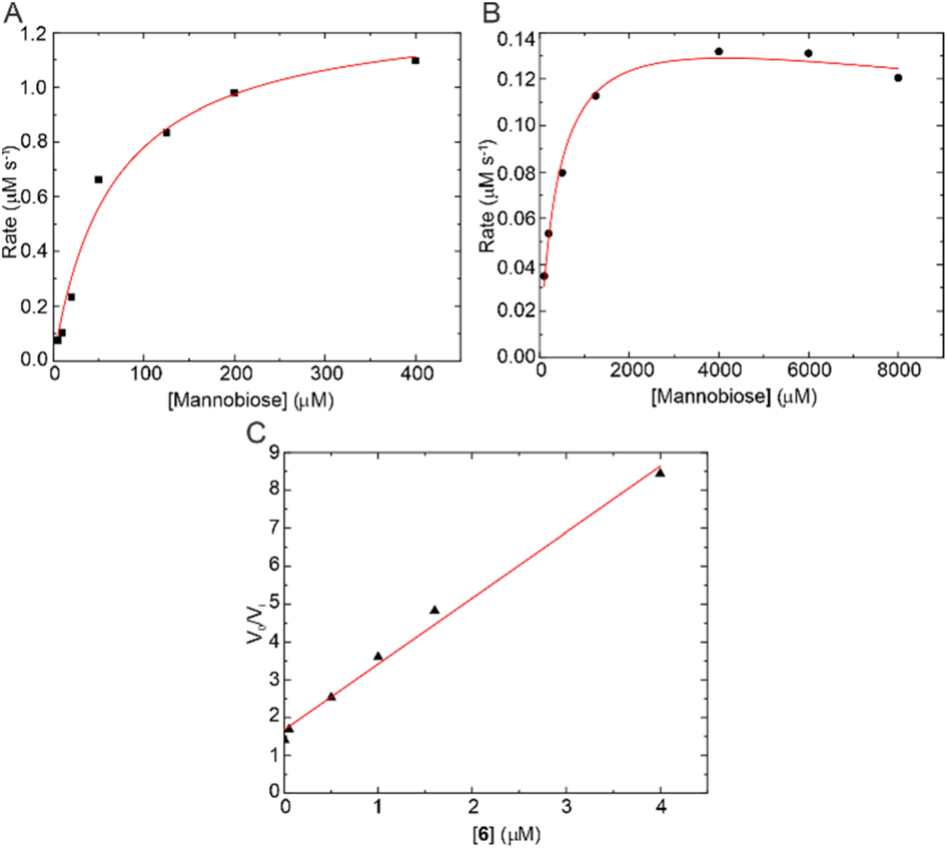
Catalytic activity of *Ck*GH47 WT compared to the *Ck*GH47 L310S mutant. (A) *Ck*GH47 Michaelis–Menten kinetics (fitting model) over a range of α-1,2-mannobiose concentrations. (B) *Ck*GH47 L310S Michaelis–Menten kinetics over a range of α-1,2-mannobiose concentrations fitted to a substrate inhibition model. (C) The *K*_i_, derived from the gradient (1/*K*_i_), for 6 against *Ck*GH47 L310S.

The L310S variant was created and shown to be catalytically viable in the degradation of α-1,2-mannobiose (Fig. 4A, B and Table S1†). The *V*_max_ value of 0.15 μM s^−1^ and the *K*_M_ value of 403 μM for the mutant were 10× and 6× lower than those of the WT.^32,33^ Similar loss in enzyme catalytic activity has also been observed in protein kinase bump and hole approaches.^25,26^

**Fig. 5:**
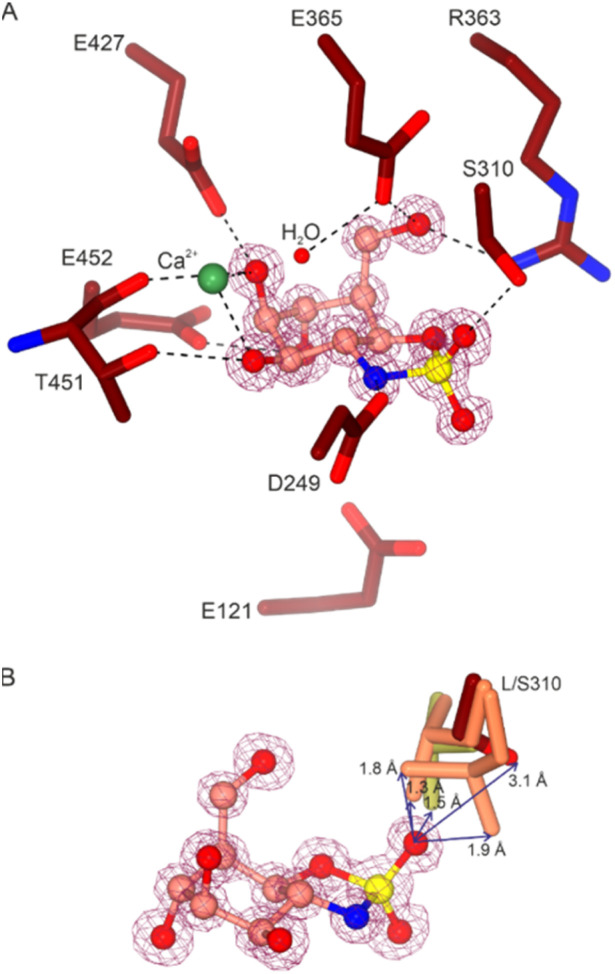
Structure of *Ck*GH47 L310S in complex with 6. (A) Active site residues (dark red) within hydrogen bonding distance to 6 (pink) in the −1 subsite. The maximum-likelihood/σA-weighted 2*F*_obs_ − *F*_calc_ map, shown in purple, is contoured at 1.3 e Å^−3^. (B) Superposition of *Ck*GH47 WT crystal structures highlighting the flexibility of residue 310; native leucine is shown in gold (PDB 4AYO) and *Ck*GH47 in complex with kifunensine is shown in orange; there are 2 alternate conformations of Leu at 50% occupancy each (PDB 5NE5).

Building on this kinetic assay, we obtained an inhibition constant by pre-incubation of the enzyme and 6 for 30 minutes and then following the same time-point procedure as for the Michaelis–Menten assay. Following the approach described by Suits *et al.*,^34^ a 574 nM *K*_i_ was obtained for 6 with the L310S mutant specifically (Fig. 4C and Table S1†). Similarly, a dissociation constant of 970 nM was obtained using isothermal titration calorimetry (Fig. S6†). To understand the interactions between 6 and *Ck*GH47 L310S regarding the introduced hole, a crystal structure complex was obtained (statistics in Table S2†). *Trans*-cyclic sulfamidate 6 binds similarly to other conformationally restricted GH47 inhibitors, with additional interactions between the sulfamidate and active site residues, for example, the nitrogen and oxygen, and D249 and R363 side chains. Additionally, the introduced serine residue is indeed now in a position to hydrogen bond to the oxygen of the sulfamidate with a distance of 3.1 Å. Also validating the initial design hypothesis, overlaying the L310S structure with 6 with that of the WT *Ck*GH47 native enzyme revealed 1.3–1.9 Å steric clashes of 6 to leucine 310 (Fig. 5B and S5†).

Finally, selectivity *versus* a panel of related exo-mannosidases was investigated. We first tested inhibition against all five human GH38 retaining α-mannosidases in overexpressed cell lysates using 4-methylumbelliferyl-α-mannoside (4-MU-α-man): Golgi mannosidase II and IIx (MAN2A1 and MAN2A2), cytosolic MAN2C1 and lysosomal MAN2B1 and MAN2B2. This α-mannosidase family follows a different conformational itinerary than GH47: ^0^*S*_2_ → *B*_2,5_ → ^1^*S*_5_.^35^ Cyclic sulfamidate 6 showed no inhibition of this panel of enzymes up to 1 mM with the exception of MAN2C1, for which an apparent IC_50_ value of 3.5 μM was observed when using cobalt as the metal ion (Table 1). Intriguingly, no IC_50_ value could be determined for MAN2C1 when the buffer was supplemented with 1 mM ZnCl_2_ instead of CoCl_2_, and compound 6 showed no activity in the other tested GH38 α-mannosidases when the buffer was enriched with CoCl_2_. Of note, the presence of cobalt has been associated with conformational changes in the active site of some GH38 α-mannosidases, which might affect the binding interaction with inhibitors.^36^ Nevertheless, additional research is required to explore which specific metal ion is present in the MAN2C1 active site in its *in vivo* state. As expected, when looking at the representative GH2 α-mannosidases *Bacteroides thetaiotaomicron Bt*Man2A, compound 6 was also inactive at 1 mM concentration.

**Table 1:** IC_50_ values for *in vitro* inhibition of GH38 retaining α-mannosidases using overexpressed cell lysates of Golgi mannosidase II and IIx (MAN2A1 and MAN2A2), cytosolic MAN2C1 and lysosomal MAN2B1 and MAN2B2 and GH2 retaining β-mannosidase using purified *Bacteroides thetaiotaomicron Bt*Man2A

Enzyme	Species	IC_50_
**GH38 exo-α-mannosidases**		
MAN2A1 (Zn^2+^)	Human	>1 mM
MAN2A2 (Zn^2+^)	Human	>1 mM
MAN2B1 (Zn^2+^)	Human	>1 mM
MAN2B2 (Zn^2+^)	Human	>1 mM
MAN2C1 (Co^2+^)	Human	3.5 ± 1.2 μM
MAN2C1 (Zn^2+^)	Human	>1 mM

**GH2 exo-β-mannosidase**		
*Bt*Man2A	Bacterial	>1 mM

## Conclusions

“Isoform-specific” inhibition of single members of multigene families is a major challenge in chemical glycobiology. Here we use the dual approach of conformational mimicry with bump and hole to establish a nanomolar inhibitor–enzyme pair: 1,6-*trans*-cyclic sulfamidate 6 and an L310S variant that allows selective inhibition of this specific α-1,2-mannosidase. Importantly, the leucine residue is conserved across all seven human ER and Golgi α-1,2-mannosidases (Fig. S8†), thus, this work lays the foundation for targeted enzyme inactivation campaigns for α-1,2-mannosidases to understand the roles of the different inverting GH47 α-1,2-mannosidases in cellular biology and disease.^37–39^

## Data availability

The authors declare that all data supporting the findings of this study are available within the article and ESI,† and raw data files are available from the corresponding authors upon request.

## Author contributions

MA, CR and GJD conceived and designed the experiments. KK, SvdB, TB and MA synthesized inhibitors under the guidance of MA, GAvdM, JDCC and HSO. ANH performed *ab initio* metadynamics calculations under the supervision of CR. AM generated (mutant) *Ck*GH47, carried out structural studies on enzyme–inhibitor complexes and determined enzyme kinetic parameters and *K*_i_ values under the supervision of GJD. NdK performed selectivity studies under the supervision of MA and JMFGA. AM, CR, GJD and MA wrote the manuscript with input from all authors.

## Conflicts of interest

There are no conflicts to declare.

## Supplementary Material

SC-014-D3SC05016E-s001
