# Supplementary material for: Trans-cyclosulfamidate mannose-configured cyclitol allows isoform-dependent inhibition of GH47 α-d-mannosidases through a bump–hole strategy
Source: Chem Sci. 2023 Nov 17;14(46):13581–6. doi: 10.1039/d3sc05016e (PMC10685318; doi:10.1039/d3sc05016e)
Supplement: SC-014-D3SC05016E-s001 [file SC-014-D3SC05016E-s001.pdf]

Electronic Supplementary Material (ESI) for Chemical Science.
This journal is © The Royal Society of Chemistry 2023

Supporting information for

Trans-Cyclosulfamidate Mannose-Configured Cyclitol Allows Specific Allele-Dependent Inhibition of GH47 α-D-Mannosidases Through a Bump-Hole Strategy.

Alexandra Males[a],#,†, Ken Kok[b],#, Alba Nin-Hill[c], Nicky de Koster[b], Sija van den Beukel[b], Thomas J. M. Beenakker[d], Gijsbert A. van der Marel[d], Jeroen D. C. Codée[d], Johannes M. F. G. Aerts[b], Herman S. Overkleeft[d], Carme Rovira[c], Gideon J. Davies[a]* and Marta Artola[b]**

[a] Department of Chemistry, University of York, YO10 5DD, United Kingdom.

[b] Department of Medical Biochemistry, Leiden Institute of Chemistry, Leiden University, Einsteinweg 55, 2333 CC, Leiden, The Netherlands.

[c] Departament de Química Inorgànica i Orgànica (Secció de Química Orgànica) and Institut de Química Teòrica i Computacional (IQTCUB), Universitat de Barcelona, Martí i Franquès 1, 08028 Barcelona, Spain, and Fundació Catalana de Recerca i Estudis Avançats (ICREA), Passeig Lluís Companys 23, 08010 Barcelona, Spain.

[d] Department of Bio-organic Synthesis, Leiden Institute of Chemistry, Leiden University, Einsteinweg 55, 2333 CC Leiden, The Netherlands.

†Current address: Biomolecular Sciences Research Centre, Sheffield Hallam University, Howard Street, Sheffield, S1 1WB.

Table of Contents

1. Supporting figures and tables S3
2. Materials and Methods S10
   2.1 Biochemical and biological methods S10
      2.1.1 Inhibition and Kinetics assays S10
      2.1.2 Isothermal titration calorimetry S10
   2.2 Crystallographic data collection and refinement statistics S10
   2.3 GH38 α-mannosidases and GH2 β-mannosidase inhibition S11
3. Chemical synthesis S13
   3.1 General experimental details S13
   3.2 Synthetic scheme for compounds **4** and **5** S14
   3.3 Synthesis and characterization of compounds **4**-**6** S14
      3.3.1 Synthesis and characterization data of **4** S14
      3.3.2 Synthesis and characterization data of **5** S16
      3.3.3 Synthesis and characterization data of **6** S17
4. NMR Spectra S19
5. References S31

1. Supporting Figures & Tables

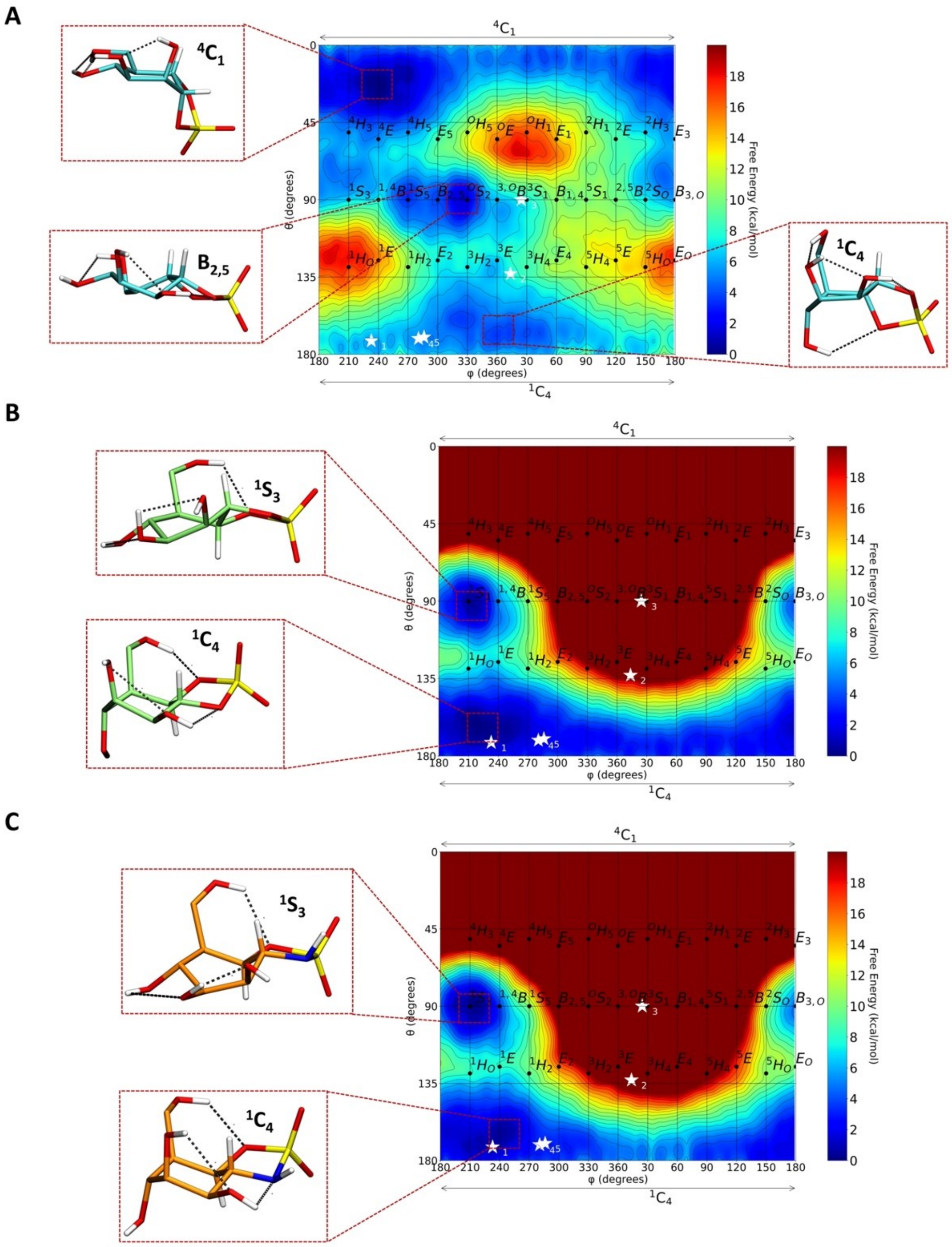

Figure S1. Intramolecular hydrogen bond interactions (shown as black dashed lines) that stabilize the main conformations of A) 1,6-*Cis*-manno-cyclosulfate (C atoms shown in cyan), B) 1,6-*Trans*-manno-cyclosulfate (C atoms shown in lime) and C) 1,6-*Trans*-manno-cyclosulfamidate (C atoms shown in orange). For visualization purposes, only polar hydrogens and the hydrogens attached to C1 and C6 are depicted.

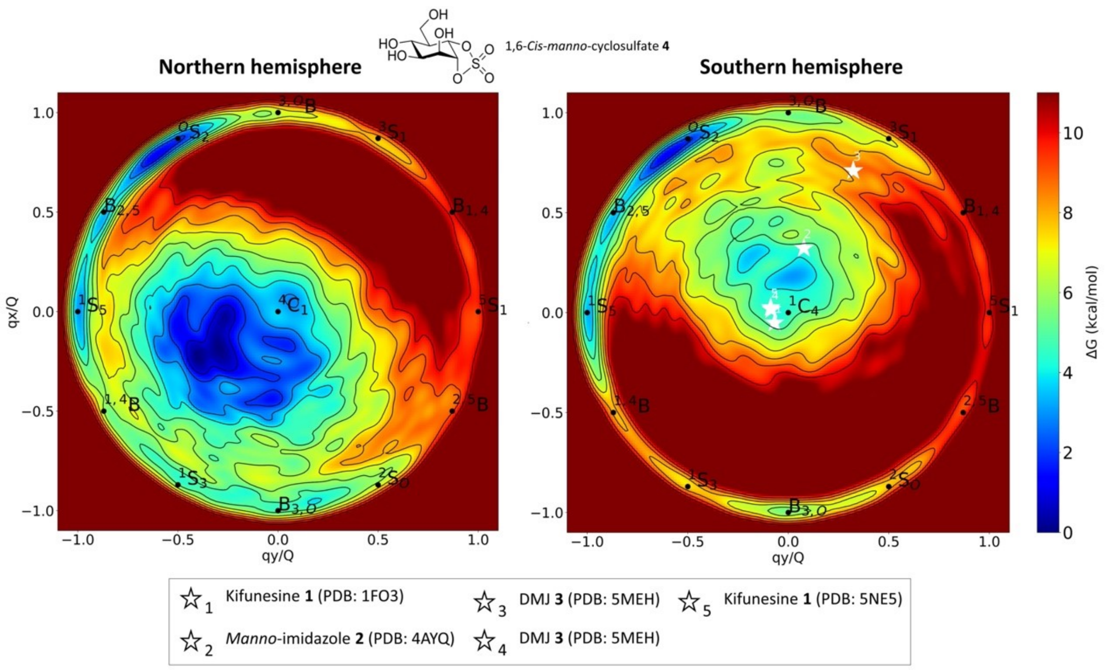

Figure S2. Conformational free energy landscapes (FELs, Stoddard projection in both northern and southern hemisphere), contoured at 1 kcal·mol^{-1} of 1,6-*Cis-manno*-cyclosulfate **4**. Observed conformations of inhibitors bound to GH47 enzymes are indicated by stars. Kifunesine (**1**) from PDB 1FO3[1] and 5NE5[2], *manno*-imidazole (**2**) from PDB 4AYQ[3] and DMJ (**3**) from PDB 5MEH.

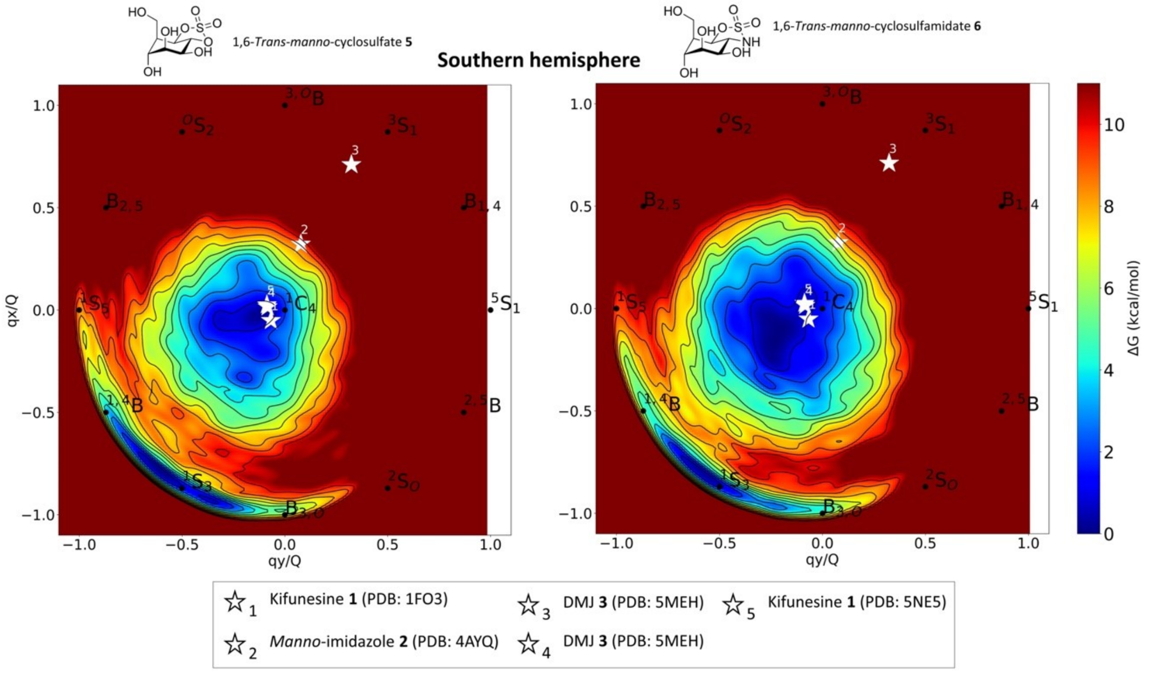

Figure S3. Conformational free energy landscapes (FELs, Stoddard projection in both northern and southern hemisphere), contoured at 1 kcal·mol^{-1} of 1,6-*Trans-manno*-cyclosulfate **5** (left) and 1,6-*Trans-manno*-cyclosulfamidate **6** (right). T Observed conformations of inhibitors bound to GH47 enzymes are indicated by stars. Kifunesine (**1**) from PDB 1FO3[1] and 5NE5[2], *manno*-imidazole (**2**) from PDB 4AYQ[3] and DMJ (**3**) from PDB 5MEH[4].

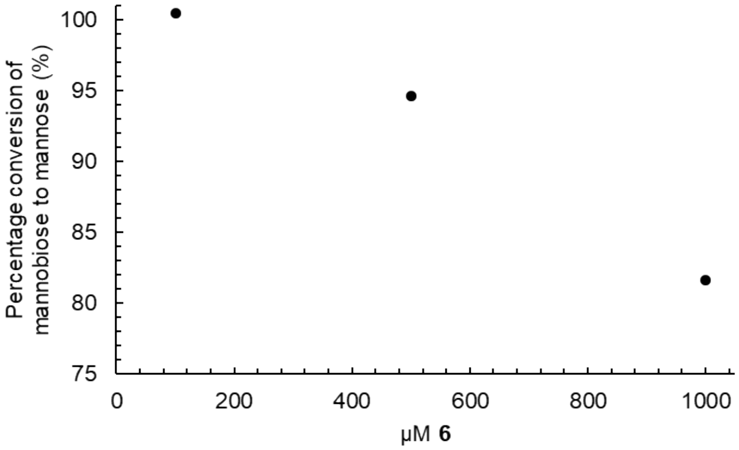

Figure S4. Inhibition of *Ck*GH47 by **6** monitored by the enzymatic conversion of mannobiose to mannose. 3 concentrations of the inhibitor were incubated with 200 nM of *Ck*GH47 for 45 minutes at 22 °C, subsequently, 189 μM of α-1,2-mannobiose was added and the reaction was incubated for 1 hour at 22 °C. The enzyme was heat denatured (90 °C for 5 minutes). Using the mannose detection kit, NADPH production at 340 nm was measured and converted to mannose concentration using a standard curve of mannose.

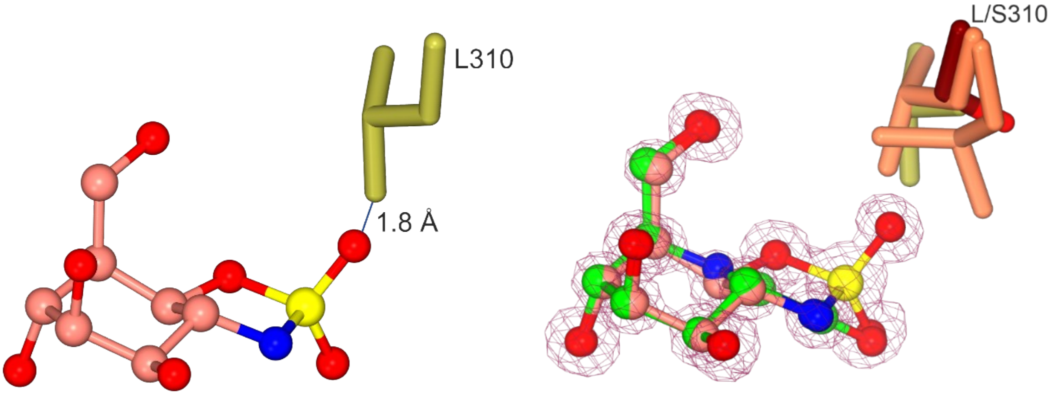

Figure S5. Docking of **6**, in pink, into the crystal structure of *Caulobacter* GH47 (PDB ID: 4AYO), in gold, showing the distance between the oxygen of **6** and L310. Left: **6** was docked into the *Ck*GH47 enzyme active site by least squares fit superposition in coot against the *Ck*GH47 and kifunensine complex (PDB ID 5NE5). Right: Position of *Ck*GH47 WT crystal structures of the inhibitors **6** (pink) and kifunensine (green); native is shown in gold (PDB ID: 4AYO) and *Ck*GH47 in complex with kifunensine is shown in orange (PDB ID: 5NE5); there are 2 alternate conformations of leucine each with 50% occupancy in the crystal structure.

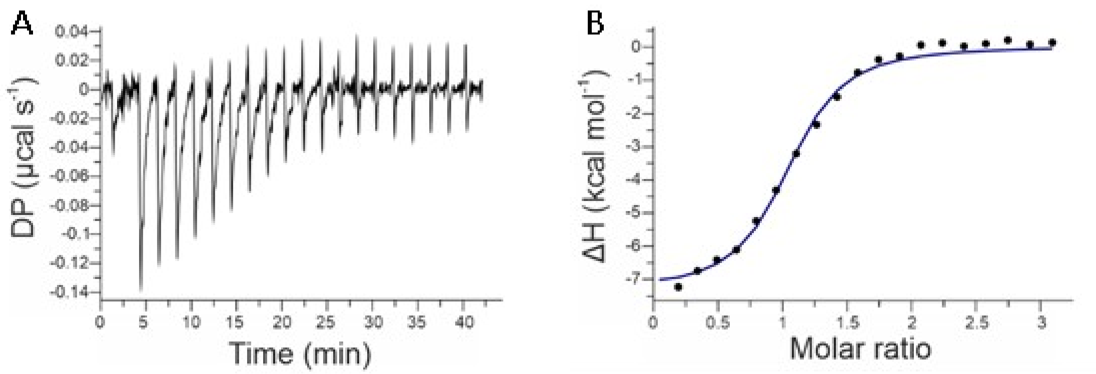

Figure S6. Isothermal titration calorimetry binding thermodynamics between *Ck*GH47 L310S and **6**. A. raw data injection profile; B. Titration curve. *N* = 1.01 ± 0.02 sites, ΔH = -7.39 ± 0.19 kcal mol^{-1}.

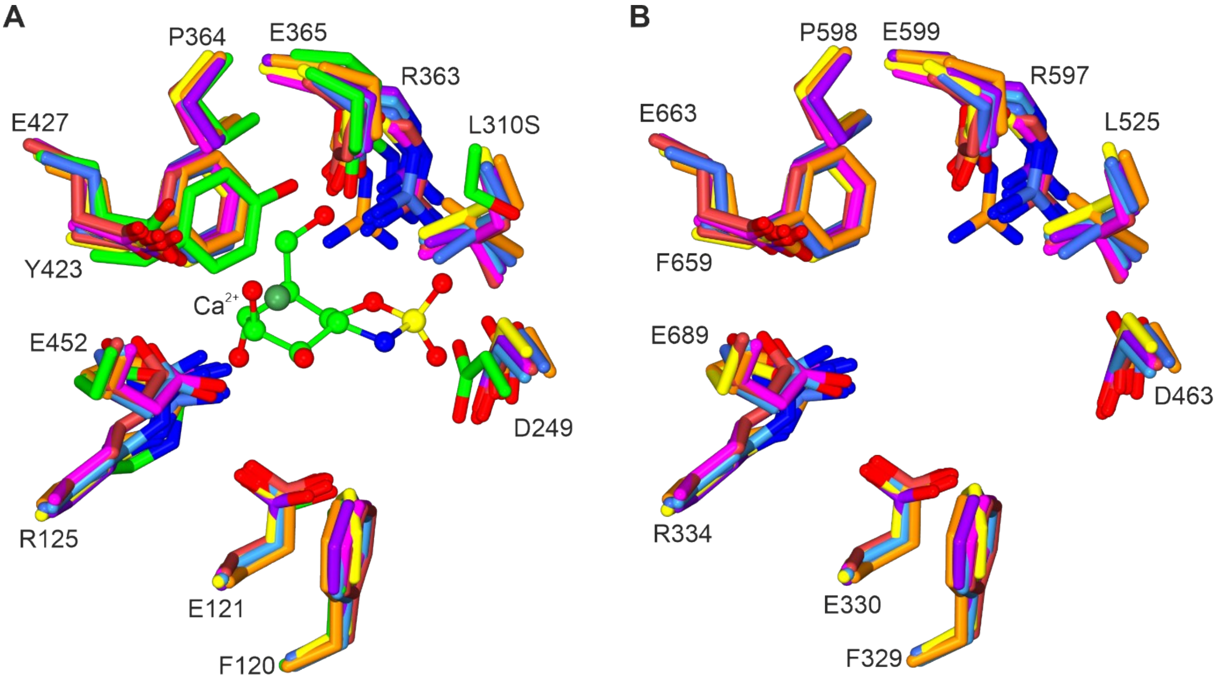

Figure S7. Overlay of the human GH47 α-1,2-mannosidase structures highlighting the conserved active site. Crystal structure of ERManI is in orange (PDB ID: 1FMI); the remaining structures were predicted by Alphafold[5], in light blue is GMIB, in dark blue is GMIA, in purple is GMIC, in yellow is EDEM2, in red is EDEM3 and in pink is EDEM1. A. The crystal structure from this work of *Ck*GH47 and **3** is in green. Residues annotated according to the numbering of *Ck*GH47. B. The human GH47 enzymes are superposed minus the *Ck*GH47 enzyme; the residues are annotated according to the numbering of *Hs*ERManI.

```
CkGH47    --------------------------------------------------------------------------------   0
HsEDEM3   ---------------------MSEAGGRGCGSPVPQRAR-----------------------------------------  18
HsEDEM1   --------------------------MQWRALVLGLVLL-----------------------------------------  13
HsEDEM2   -------------------------------------MP-----------------------------------------   2
HsERManI  MAACEGRRSGALGSSQSDFLTPPVGGAPWAVATTVVMYPPPPPPPHRDFISVTLSFGESYDNSKSWRRRSCWRKWKQLSR  80
HsGMIC    --------------MLMRKVPGFVPASPWGLRLPQKFLF-----------------------------------------  25
HsGMIA    -----------------------MNSNFITFDLKMSLLP-----------------------------------------  16
HsGMIB    MTTPALLPLSGRRIPPLNLGPPSFPHHRATLRLSEKFIL-----------------------------------------  39

CkGH47    -----------------------MTASHLSRRHAL---------------------------------------------  12
HsEDEM3   -----WRLVAATAAFC----------------------------------------------------------------  29
HsEDEM1   -----RLGLHGVLWLVFGLGPSMGFYQRFPLSFGF-----------QRLRSPDGPASPTSGPVGRPGGVSGPSWLQPPGT  77
HsEDEM2   -----FRLLIPLGLLC----------------------------------------------------------------  13
HsERManI  LQRNMILFLLAFLLFC-GLLFYINLADH-WKALAFRLEE------EQKMRPEIAGLKPANPPVLPAPQKADTDPENLPEI 152
HsGMIC    -----LLFLSGLVTLCFGALFLLPHSSR-LKRLFL------------APRTQQPGLEVVAEIAGHAPAREQEPPPNPAPA  87
HsGMIA    -----SNLFSAFITLCFGAIFFLPDSSKLLSGVLFHSSPALQPAADHKPG---PGARAEDAAEGRARRREEGAPGDPEAA  88
HsGMIB    -----LLILSAFITLCFGAFFFLPDSSK-HKRFDLGLEDVLIPHVDAGKGAKNPGVFLIHGPDEHRHREEE--------- 104

CkGH47    ---------------------------------ALVASAAAAPAFAAETTP-----------------------------  30
HsEDEM3   ----------------------------------LVSATSVWTAGAEPMSR-----------------------------  46
HsEDEM1   ---------------------------------GAAQSPRKAPRRPGPGMCGPANWGYVLGGRGRGPDEYEKRYSGAFPP 124
HsEDEM2   ---------------------------------ALLPQHHGAPGPDGSA-------------------------------  29
HsERManI  SSQKTQRHIQRGPPHLQIRPPSQDLKDGTQEEATKRQEAPVDPRPEGDPQRTVISWRGAVIEPEQGTELPSRRA--EVPT 230
HsGMIC    APAPGEDDPSS-------------WASPRRRKGGLRRTRPTGPREEATAARGNSIPAS---RPGDE-GVPFRFDFNAFRS 150
HsGMIA    L----EDNLAR-------------IRENHER--ALREAKETLQKLPEEIQRDILLEKK---KVAQD-QLRDKAPFRGLPP 145
HsGMIB    -----ERLRNK-------------IRADHEK--ALEEAKEKLRKSREEIRAEIQTEKN---KVVQEMKIKENKP---LPP 158

CkGH47    ------------------EDWKALAADVRSEFQWAWQGYVAKAWGKDEINPVSGTSR------------SFFIEGHDLGL  80
HsEDEM3   ------------------EEKQKLGNQVLEMFDHAYGNYMEHAYPADELMPLTCRGRVRGQ-EPSRGDVDDALGKF--SL 105
HsEDEM1   ------------------QLRAQMRDLARGMFVFGYDNYMAHAFPQDELNPIHCRGRGPDRGDPSNLNINDVLGNY--SL 184
HsEDEM2   ------------------PDPAHYRERVKAMFYHAYDSYLENAFPFDELRPLTCDGH-------------DTWGSF--SL  76
HsERManI  KPPLPPARTQGTP-----VHLNYRQKGVIDVFLHAWKGYRKFAWGHDELKPVSRS-------------FSEWFGL---GL 289
HsGMIC    RLRHPVLGTRADESQEPQSQVRAQREKIKEMMQFAWQSYKRYAMGKNELRPLTKDGY-----------EGNMFGGLS-GA 218
HsGMIA    VDFVPPIGVESREPAD--AAIREKRAKIKEMMKHAWNNYKGYAWGLNELKPISKGGH-----------SSSLFGNIK-GA 211
HsGMIB    VPIPNLVGIRGGDPED--NDIREKREKIKEMMKHAWDNYRTYGWGHNELRPIARKGH-----------SPNIFGSSQMGA 225
                                         :  .: .*   .   :*: *:                          .

CkGH47    SLVEALDTLWIMGLDAEFQAGVDWVKANLSFDVDGNAQVFETNIRLVGGLLSAHLAS--------------GDPVLLAKA 146
HsEDEM3   TLIDSLDTLVVLNKTKEFEDAVRKVLRDVNLDNDVVVSVFETNIRVLGGLLGGHSLAIMLKEKGE--YMQWYNDELLQMA 183
HsEDEM1   TLVDALDTLAIMGNSSEFQKAVKLVINTVSFDKDSTVQVFEATIRVLGSLLSAHRIITDSKQPFGDMTIKDYDNELLYMA 264
HsEDEM2   TLIDALDTLLILGNVSEFQRVVEVLQDSVDFDIDVNASVFETNIRVVGGLLSAHLLSKKAGVEVEAGWPCSGP--LLRMA 154
HsERManI  TLIDALDTMWILGLRKEFEEARKWVSKKLHFEKDVDVNLFESTIRILGGLLSAYHLS--------------GDSLFLRKA 355
HsGMIC    TVIDSLDTLYLMELKEEFQEAKAWVGESFHLNVSGEASLFEVNIRYIGGLLSAFYLT--------------GEEVFRIKA 284
HsGMIA    TIVDALDTLFIMEMKHEFEEAKSWVEENLDFNVNAEISVFEVNIRFVGGLLSAYYLS--------------GEEIFRKKA 277
HsGMIB    TIVDALDTLYIMGLHDEFLDGQRWIEDNLDFSVNSEVSVFEVNIRFIGGLLAAYYLS--------------GEEIFKIKA 291
          :::::***: ::    **      :   . :. .   .:** .** :*.**...                     :   *

CkGH47    RDLADRLAKAFEASPHGLPWRYVNLRTG-------AVSDPETNLAEIGTYLSEFGVLSQLTGERKYFDMAKRAMRHTLDR 219
HsEDEM3   KQLGYKLLPAFN-TTSGLPYPRINLKFGIRKPEARTGTETDTCTACAGTLILEFAALSRFTGATIFEEYARKALDFLWEK 262
HsEDEM1   HDLAVRLLPAFENTKTGIPYPRVNLKTGVP-----PDTNNETCTAGAGSLLVEFGILSRLLGDSTFEWVARRAVKALWNL 339
HsEDEM2   EEAARKLLPAFQ-TPTGMPYGTVNLLHGVN-----PGETPVTCTAGIGTFIVEFATLSSLTGDPVFEDVARVALMRLWES 228
HsERManI  EDFGNRLMPAFR-TPSKIPYSDVNIGTGVAHPPRWTS---DSTVAEVTSIQLEFRELSRLTGDKKFQEAVEKVTQHIHGL 431
HsGMIC    IRLGEKLLPAFN-TPTGIPKGVVSFKSG---NWGWATAGSSSILAEFGSLHLEFLHLTELSGNQVFAEKVRNIRKVLRKI 360
HsGMIA    VELGVKLLPAFH-TPSGIPWALLNMKSGIGRNWPWASGG-SSILAEFGTLHLEFMHLSHLSGNPIFAEKVMNIRTVLNKL 355
HsGMIB    VQLAEKLLPAFN-TPTGIPWAMVNLKSGVGRNWGWASAG-SSILAEFGTLHMEFIHLSYLTGDLTYYKKVMHIRKLLQKM 369
             . .*  **  :   :*   :.:  *       .     :  *   :   **  *: : *   :   .

CkGH47    R-SKIGLMAANIHAMTGAFTSRNA-SIDVYADSFYEYLWDAWALFG--DEDCKRWAVECVDAQLAHQAKR-------YDG 288
HsEDEM3   RQRSSNLVGVTINIHTGDWVRKDS-GVGAGIDSYYEYLLKAYVLLG--DDSFLERFNTHYDAIMRYIS-----------Q 328
HsEDEM1   RSNDTGLLGNVVNIQTGHWVGKQS-GLGAGLDSFYEYLLKSYILFG--EKEDLEMFNAAYQSIQNYLRRGREACNEGEGD 416
HsEDEM2   R-SDIGLVGNHIDVLTGKWVAQDA-GIGAGVDSYFEYLVKGAILLQ--DKKLMAMFLEYNKAIRNYTRFD---------- 294
HsERManI  SGKKDGLVPMFINTHSGLFTHLGVFTLGARADSYYEYLLKQWIQGGKQETQLLEDYVEAIEGVRTHLLRH--------SE 503
HsGMIC    E-KPFGLYPNFLSPVSGNWVQHHV-SVGGLGDSFYEYLIKSWLMSGKTDMEAKNMYYEALEAIETYLLNV---------S 429
HsGMIA    E-KPQGLYPNYLNPSSGQWGQHHV-SVGGLGDSFYEYLLKAWLMSDKTDLEAKKMYFDAVQAIETHLIRK---------S 424
HsGMIB    D-RPNGLYPNYLNPRTGRWGQYHT-SVGGLGDSFYEYLLKAWLMSDKTDHEARKMYDDAIEAIEKHLIKK---------S 438
               .*    :   :* :       :.   **::*** .        : .         ..   :

CkGH47    RLWFPMVDFETGAVTG-TAQSELAAYYAGLLGQVGR----------KAQGDDYLASF--TYLQATFGVIPESIDVT---- 351
HsEDEM3   PPLLLDVHIHKPMLNARTWMDALLAFFPGLQVLKGD----------IRPAIETHEMLYQVIKK--HNFLPEAFTTD---- 392
HsEDEM1   PPLYVNVNMFSGQLMN-TWIDSLQAFFPGLQVLIGD----------VEDAICLHAFYYAIWKR--YGALPERYNWQ---- 479
HsEDEM2   -DWYLWVQMYKGTVSM-PVFQSLEAYWPGLQSLIGD----------IDNAMRTFLNYYTVWKQ--FGGLPEFYNIP---- 356
HsERManI  PSKLTFVGELAHGRFS-AKMDHLVCFLPGTLALGVY---HGLPASHMELAQELMETCYQMNRQMETGLSPEIVHFNLYPQ 579
HsGMIC    PGGLTYIAEWRGGILD-HKMGHLACFSGGMIALGAEDAKEEKRAHYRELAAQITKTCHESYARSDTKLGPEAFWFN---- 504
HsGMIA    SSGLTYIAEWKRGLLE-HKMGHLTCFAGGMFALGADAAPEGMAQHYLELGAEIARTCHESYNRTFMKLGPEAFRFD---- 499
HsGMIB    RGGLTFIGEWKNGHLE-KKMGHLACFAGGMFALGADGSRADKAGHYLELGAEIARTCHESYDRTALKLGPESFKFD---- 513
                :               * .:  *                    .            .      **
```

```
CkGH47    ------TGQPRRKHTGLRPEYPDACLNLWLIDRDPRYRRLAAIHYREMKATSRAA-FGYTALKDIT-TRPMTQDDNCPGY 423
HsEDEM3   -------FRVHWAQHPLRPEFAESTYFLYKATGDPYYLEVGKTLIENLNKYARVP-CGFAAMKDV---RTGSHEDRMDSF 461
HsEDEM1   ------LQAPDVLFYPLRPELVESTYLLYQATKNPFYLHVGMDILQSLEKYTKVK-CGYATLHHV---IDKSTEDRMESF 549
HsEDEM2   ----QGYTVEKREGYPLRPELIESAMYLYRATGDPTLLELGRDAVESIEKISKVE-CGFATIKDL---RDHKLDNRMESF 428
HsERManI  PGRRDVEVKPADRHNLLRPETVESLFYLYRVTGDRKYQDWGWEILQSFSRFTRVPSGGYSSINNVQDPQKPEPRDKMESF 659
HsGMIC    SGREAVATQLSESYYILRPEVVESYMYLWRQTHNPIYREWGWEVVLALEKYCRTE-AGFSGIQDVY-SSTPNHDNKQQSF 582
HsGMIA    GGVEAIATRQNEKYYILRPEVMETYMYMWRLTHDPKYRKWAWEAVEALENHCRVN-GGYSGLRDVY-LLHESYDDVQQSF 577
HsGMIB    GAVEAVAVRQAEKYYILRPEVIETYWYLWRFTHDPRYRQWGWEAALAIEKYCRVN-GGFSGVKDVY-SSTPTHDDVQQSF 591
                          ****  ::   ::    :      .      :.   ..   *:: :. :         :   .:

CkGH47    WWSEQMKYYYLLFSDTPRIDYGQ--------------------LQLSTEANVLRGFRKV--------------------- 462
HsEDEM3   FLAEMFKYLYLLFADKEDIIFDIED------------------YIFTTEAHLLPLWLSTTNQSISKKNTTSEYTELDDSN 523
HsEDEM1   FLSETCKYLYLLF-DEDNPVHKSGTR-----------------YMFTTEGH-----IVSVDEHLREL------------- 593
HsEDEM2   FLAETVKYLYLLF-DPTNFIHNNGSTFDAVITPYGECILGAGGYIFNTEAHPIDPAALHCCQRLKEE------------- 494
HsERManI  FLGETLKYLFLLFSDDPNLLSLDA-------------------YVFNTEAHPLPI------------------------- 695
HsGMIC    FLAETLKYLYLLFSED-DLLSLED-------------------WVFNTEAHPLPVNHSDSSGR----------------- 625
HsGMIA    FLAETLKYLYLIFSDD-DLLPLEH-------------------WIFNSEAHLLPILPKDKKEV----------------- 620
HsGMIB    FLAETLKYLYLLFSGD-DLLPLDH-------------------WVFNTEAHPLPVLHLANTTL----------------- 634
          : .*  ** :*:*                                :.:*.:

CkGH47    -------------------------------------------------------------------------------- 462
HsEDEM3   FDWTCPNTQILFPNDPLYAQSIREPLKNVVDKSCPRGIIRVEESFRSGAKPPLRARDFMATNPEHLEILKKMGVSLIHLK 603
HsEDEM1   -PW----------------------------------------------------------------------------- 595
HsEDEM2   -QWEVED------------------------------------------------------------------------- 500
HsERManI  --W----------------------------------------------------------------------------- 696
HsGMIC    -AW----------------------------------------------------------------------------- 627
HsGMIA    -EI----------------------------------------------------------------------------- 622
HsGMIB    -SG----------------------------------------------------------------------------- 636

CkGH47    -------------------------------------------------------------------------------- 462
HsEDEM3   DGRVQLVQHAIQAASSIDAEDGLRFMQEMIELSSQQQKEQQLPPRAVQIVSHPFFGRVVLTAGPAQFGLDLSKHKETRGF 683
HsEDEM1   --------------------------KEFFSEEGGQDQ----------------GGKSVHRPKPHE-------------- 619
HsEDEM2   ------------------------LMREFYSLKRSRSK----------------FQKNTVSSGPWE-------------- 526
HsERManI  --------------------------------------------------------------TPA--------------- 699
HsGMIC    --------------------------------------------------------------GRH--------------- 630
HsGMIA    --------------------------------------------------------------REE--------------- 625
HsGMIB    --------------------------------------------------------------NPAVR------------- 641

CkGH47    -------------------------------------------------------------------------------- 462
HsEDEM3   VASSKPSNGCSELTNPEAVMGKIALIQRGQCMFAEKARNIQNAGAIGGIVIDDNEGSSSDTAPLFQMAGDGKDTDDIKIP 763
HsEDEM1   ---------------LKV----I------------------------------NSSSNCNRVP-------DER--RYSLP 641
HsEDEM2   ---------------PPARPGTL------------------------------FSPENHDQAR-------ERKPAKQKVP 554
HsERManI  -------------------------------------------------------------------------------- 699
HsGMIC    -------------------------------------------------------------------------------- 630
HsGMIA    -------------------------------------------------------------------------------- 625
HsGMIB    -------------------------------------------------------------------------------- 641

CkGH47    -------------------------------------------------------------------------------- 462
HsEDEM3   MLFLFSKEGSIILDAIREYEEVEVLLSDKAKDRDPEMENEEQPSSENDSQNQSGEQISSSSQEVDLVDQESSEENSLNSH 843
HsEDEM1   LKSIYM-------------------------------------------------------------------------- 647
HsEDEM2   LLSCPS-------------------------------------------------------------------------- 560
HsERManI  -------------------------------------------------------------------------------- 699
HsGMIC    -------------------------------------------------------------------------------- 630
HsGMIA    -------------------------------------------------------------------------------- 625
HsGMIB    -------------------------------------------------------------------------------- 641

CkGH47    -------------------------------------------------------------------------------- 462
HsEDEM3   PESLSLADMDNAASISPSEQTSNPTENHETTNLNGECTDLDNQLQEQSETEEDSNPNVSWGKKVQPIDSILADWNEDIEA 923
HsEDEM1   ----------------------------------------------------------------RQIDQMVG-------L 656
HsEDEM2   ----------------------------------------------------------------QPFTSKLA-------L 569
HsERManI  -------------------------------------------------------------------------------- 699
HsGMIC    -------------------------------------------------------------------------------- 630
HsGMIA    -------------------------------------------------------------------------------- 625
HsGMIB    -------------------------------------------------------------------------------- 641

CkGH47    --------- 462
HsEDEM3   FEMMEKDEL 932
HsEDEM1   I-------- 657
HsEDEM2   LGQVFLDSS 578
HsERManI  --------- 699
HsGMIC    --------- 630
HsGMIA    --------- 625
HsGMIB    --------- 641
```

Figure S8. Sequence alignment of the seven GH47 human enzymes and *Ck*GH47 using MUSCLE multiple sequence alignment.[6] L310 is highlighted in bold with a yellow background.

Table S1. Catalytic activity of WT and L310S *Caulobacter* GH47 mannosidase.

	*Ck*GH47 WT	*Ck*GH47 L310S
V_{max} (μM s^{-1})	1.29 ± 0.09	0.154 ± 0.01
K_M (μM)	65.7 ± 14.2	402.8 ± 71.3
k_{cat} (s^{-1})	6.45	0.308
k_{cat}/K_M ($μM^{-1}$ s^{-1})	0.098	0.0076
K_i α-1,2-mannobiose (mM)		42.7 ± 24.8
K_i **6** (nM)	n.d.	574
K_D **6** (nM)	n.d.	970 ± 158

Table S2. Data statistics for data collection and structural refinement of *Ck*GH47 in complex with **6**.

	*Ck*GH47
Ligand	**6**
PDB code	**8B5M**
**Data collection**	
Wavelength (Å)	0.799
Space group	*H*3
Cell dimensions:	
a, *b*, *c* (Å)	144.13, 144.13, 50.32
α, β, γ (°)	90, 90, 120
Resolution (Å)	72.02-0.97 (0.99-0.97)
R_{merge}	0.16 (1.49)
R_{pim}	0.05 (0.49)
CC (1/2)	1.00 (0.61)
I / σ*I*	7.7 (1.3)
Completeness (%)	100.0 (100.0)
Redundancy	10.2 (10.0)
**Refinement**	
Resolution (Å)	72.02-0.97 (0.99-0.97)
No. reflections	230864
R_{work} / R_{free}	0.14/0.15
No. atoms	
Protein	3554
Ligands/Ions	16/3
Water	715
B-factors (Å^2)	
Protein	7
Ligand/Ions	5/4
Water	19
R.m.s. deviations	
Bond lengths (Å)	0.014
Bond angles (°)	1.9
Ramachandran	
Favoured (%)	96.5
Allowed (%)	3.0
Outliers (%)	0.5

2. Materials and Methods

2.1 Biochemical and biological methods

2.1.1 *Ck*GH47 WT and L310S mutant inhibition and kinetics assays

For all assays, *Ck*GH47 WT enzyme was diluted to 200 nM, and *Ck*GH47 L310S was diluted to 500 nM into buffer containing 50 mM HEPES pH 7.0, 100 mM NaCl and 2 mM $CaCl_2$. To obtain Michaelis-Menten kinetics for the wildtype enzyme, a range of α-1,2-mannobiose substrate concentrations from 5 to 400 µM and time points of 1, 2, 3, 4, 5 minutes were used. To obtain Michaelis-Menten kinetics for the L310S mutant enzyme, a range of α-1,2-mannobiose substrate concentrations from 100 µM to 8 mM and time points of 5, 10, 15, 20 minutes were used. Lastly, to achieve an inhibition constant, the method described by Suits and colleagues was used whereby the substrate α-1,2-mannobiose concentration (130 µM) was greatly less than the K_M ([S]<<K_M).[7] The gradient of the graph, V_0/V_i (absence and presence of inhibitor) against a range of **6** inhibitor concentrations from 5 nM to 4 µM, was $1/K_i$. Time points were taken at 5, 10, 20 and 30 minutes. After taking each time point, the sample was heat denatured at 95 °C for 5 minutes. The reactions were conducted in triplicate and averaged.

The samples were plated into a 384-well clear plate and absorbance was monitored using an Epoch microplate spectrophotometer (BioTek). The mannose detection kit (Megazyme) was used to detect the presence of mannose through a coupled assay whereby the final step, the formation of NADPH and gluconate-6-phosphate from $NADP^+$ and glucose-6-phosphate by glucose-6-phosphate dehydrogenase, was monitored at 340 nm. The absorbance after hexokinase was added to the mixture (phosphorylation of mannose producing mannose-6-phosphate) was subtracted from the final absorbance. A standard curve of absorbance against a range of mannose concentrations was used to determine the concentration of mannose produced in the enzyme reactions.

2.1.2 Isothermal titration calorimetry

*Ck*GH47 L310S and **6** were diluted into buffer containing 50 mM HEPES pH 7.0, 100 mM NaCl and 2 mM $CaCl_2$. 300 µM **6** was added by syringe to 20.4 µM *Ck*GH47 L310S in the calorimeter cell using a MicroCal ITC200 calorimeter at 25 °C over 20 injections. The experiment was duplicated to obtain a consistent K_D value. The binding affinity was obtained using the one set of sites fitting model within the MicroCal PEAK-ITC analysis software.

2.2 Crystallographic data collection and refinement of *Ck*GH47

*Ck*GH47 WT gene cloned into the pET21a vector was mutated using the Q5 site-directed mutagenesis kit (New England Biolabs) where the forward primer was 5′-ACAGAGCGAAtcgGCCGCATATTATGCGGG-3′ and the reverse primer was 5′-GCGGTACCGGTAACCGCA-3′. Protein production and purification was followed as described previously.[3] Using the hanging drop method, 12.5 mg mL^{-1} *Ck*GH47 L310S protein crystallised under the conditions 23% PEG3350, 0.1 M MES pH 7.0, 0.2 M ammonium acetate. A 20-times diluted seed stock of *Ck*GH47 WT crystals was used to aid crystallisation; the protein to reservoir to seed stock ratio was 0.5:0.4:0.1. Mature crystals were soaked with 2.5 mM **6** for 72 hrs. Structure data were collected on the Diamond I03 MX beamline. Reference data in the form of a reflections file and a Free R flag data set were copied from PDB ID: 4AYO and used in indexing and integration using AIMLESS from the CCP4i2 software suite.[3][8] Refinement of the model was conducted through cycles of REFMAC and manual model building in COOT.[9][10] Validation of the ligand conformation was conducted using PRIVATEER. Details of the data collection and refinement statistics are shown in Table **S2**. Figures of the structure were produced by using CCP4mg.

2.3 GH38 α-mannosidases and GH2 β-mannosidase inhibition studies

All chemicals were of analytical grade and purchased from Sigma-Aldrich unless stated otherwise. Optimal stability of cell homogenates and enzyme working stocks was ensured by preparation and temporary storage on ice. Cell homogenates were prepared and used on the same day. Protein concentrations were quantified using the Pierce BCA Protein Assay Kit (ThermoFisher). Protein samples were measured in clear flat-bottom polystyrene 96 well microplates (Greiner Bio-One) using a EMax Plus Microplate Reader (Molecular Devices). Activity measurements of human GH38 α-mannosidases and bacterial β-mannosidase BtMan2A were performed in black flat-bottom polystyrene 96 wells microplates (Greiner Bio-One) and total fluorescence was measured using a Perkin Elmer LS-55 fluorescence spectrometer at wavelengths λ_{ex} = 366 and λ_{em} = 445 nm, with the signal optimized for medium gain readings. The fluorescence values of each enzyme sample were corrected for the background signal of a blank sample containing no enzyme. In the case of IC_{50} determinations, values were normalized against a control sample containing enzyme without added inhibitor. For a single experiment, three separate curves were fitted from a technical triplicate by setting the inhibitor concentration in µM against the normalized response using GraphPad Prism 8.4.2 software. The Prism functions '[inhibitor] *versus* normalized response' and '[inhibitor] *versus* normalized response -- variable slope' were compared for each curve using the Akaike's Information Criterion method to select the preferred model. Reported IC_{50} values are mean ± standard deviation from a biological replicate (n=2).

General cell culture protocol
HEK293T cells were grown to confluency at 37 °C under a 5% CO_2 atmosphere in Dulbecco's Modified Eagle's Medium (DMEM) with a high glucose concentration (Capricorn Scientific, DMEM-HPXA) that was supplemented with 10% fetal calf serum, 0.1% (w/v) penicillin/streptomycin and 1% (v/v) Glutamax. Cells were passaged twice a week to new round polystyrene (PS) tissue culture dishes (Sarstedt) by detachment off the former culture plates by careful pipetting. To prepare cell pellets for activity measurements, plates were grown to confluency and harvested in phosphate buffered saline (PBS). After centrifugation at 1500 rpm for 5 min, the remaining PBS was aspirated and the cells were resuspended in fresh PBS. The concentration of the resulting suspension was then counted using a TC20 automated cell counter (BioRad). Aliquots were centrifuged at 2000 rpm for 5 min and the resulting cell pellets were stored at -80 °C until further use.

4-MU activity assay protocol for human GH38 α-mannosidases
HEK293T cells stably overexpressing human GH38 α-mannosidases MAN2A1, MAN2A2, MAN2B1, MAN2B2 and MAN2C1 were prepared as previously described[11] and were kindly donated by Dr. C. L. Kuo. Homogenates were prepared by sonicating cell pellets (Sonics VibraCell, 5x1 s with 30 s interval at 20% amplitude) in 150 mM McIlvain buffer of pH 5.5 (MAN2A1, MAN2A2), pH 4.5 (MAN2B1, MAN2B2) or pH 6.7 (MAN2C1) containing either 2 mM $ZnCl_2$ (MAN2A1, MAN2A2, MAN2B1, MAN2B2) or 2 mM $CoCl_2$ (MAN2C1). Lysates were further diluted in the same buffer in order to reach a signal of approximately 500-600 arbitrary units (Arbs) on the LS-55 spectrometer. For IC_{50} measurements, an equal volume of homogenate (12.5 µL containing MAN2A1 62.5 µg, MAN2A2 75 µg, MAN2B1 2.5 µg, MAN2B2 12.5 µg or MAN2C1 5 µg) was pre-incubated with inhibitor solution diluted in 1% DMSO in water (12.5 µL, increasing concentrations, final DMSO concentration 0.5%) at 37 °C for 30 min. Samples containing equal protein amounts (µg) of wildtype HEK239T homogenate in the corresponding buffer were included in each experiment as control for the enzyme overexpression. 4-MU substrate mix (100 µL) was then added containing 10 mM 4-MU-α-D-mannopyranoside (Glycosynth), 0.1% (w/v) bovine serum albumin (BSA) and 1 mM $ZnCl_2$ or $CoCl_2$ in 150 mM McIlvaine buffer at the corresponding pH,

and the samples were again incubated for 30 min at 37 °C. The reaction was stopped by adding 200 µL 1 M Glycine-NaOH buffer of pH 10.3 and fluorescence was measured directly after.

4-MU activity assay protocol for bacterial GH2 β-mannosidase BtMan2A

Recombinant BtMan2A enzyme was prepared as described previously[12] and was kindly donated by Dr. W. Offen from the University of York, UK. BtMan2A enzyme was diluted in 50 mM HEPES buffer of pH 8 containing 2 mM DTT and 250 mM NaCl, and was stored as 10 µM aliquots at -20 °C. A working stock for activity measurements was prepared by thawing one aliquot for 15 min on ice and subsequently diluting it with cold 150 mM McIlvaine buffer with 0.1% BSA to a final concentration of 12.5 nM. For IC_{50} measurements, an equal volume of enzyme dilution (12.5 µL, 156.25 fmol) was pre-incubated with inhibitor solution diluted in 1% DMSO in water (12.5 µL, increasing concentrations, final DMSO concentration 0.5%) at 37 °C for 30 min. 4-MU substrate mix (100 µL) was then added containing 10 mM 4-MU-β-ᴅ-mannopyranoside (Glycosynth) and 0.1% (w/v) BSA in 150 mM McIlvaine buffer at pH 5.6, and the samples were again incubated for 30 min at 37 °C. The reaction was stopped by adding 200 µL 1 M Glycine-NaOH buffer of pH 10.3 and fluorescence was measured directly after.

3. Chemical Synthesis

3.1 General experimental details

All reagents were of experimental grade and were used without further purification unless stated otherwise. Dichloromethane (DCM) and tetrahydrofuran (THF) were stored over 3 Å molecular sieves and *N,N*-dimethylformamide (DMF) was stored over 4 Å molecular sieves, which were dried *in vacuo* before use. All reactions were performed under an N_2 atmosphere unless stated otherwise. Reactions were monitored by analytical thin layer chromatography (TLC) using Merck aluminum sheets pre-coated with silica gel 60 with detection by UV-absorption (254 nm) and by spraying with a solution of $(NH_4)_6Mo_7O_{24}\cdot H_2O$ (25 g/L) and $(NH_4)_4Ce(SO_4)_4\cdot H_2O$ (10 g/mL) in 10% sulfuric acid followed by charring at ~150 ℃ or by spraying with an aqueous solution of $KMnO_4$ (7%) and K_2CO_3 (2%) followed by charring at ~150 ℃. Column chromatography was performed manually using either Baker or Screening Device silica gel 60 (0.04-0.063 mm) or a Biotage Isolera™ flash purification system using silica gel cartridges (Screening Device SilicaSep HP, particle size 15-40 µm, 60A) in the indicated solvents. ^{1}H-NMR and ^{13}C-NMR spectra were recorded on Bruker AV-500 (500/126 MHz), and Bruker AV-400 (400/101 MHz) spectrometer in the given solvent. Chemical shifts are given in ppm (δ) relative to the chloroform, methanol or dimethylsulfoxide residual solvent peak or tetramethylsilane (TMS) as internal standard. All given ^{13}C-NMR spectra are proton decoupled. The following abbreviations are used to describe peak patterns when appropriate: s (singlet), d (doublet), t (triplet), q (quartet), m (multiplet), Ar (aromatic), C_q (quarternary carbon). 2D NMR experiments (HSQC, COSY) were carried out to assign protons and carbons of the new structures and numbering and assignation follows the general numbering shown in compound **14** (See synthesis and characterization section). High-resolution mass spectra (HRMS) of compounds were recorded with an LTQ Orbitrap (Thermo Finnigan) equipped with an electronspray ion source in positive mode (source voltage 3.5 kV, sheath gas flow 10 mL/min, capillary temperature 250 ℃) with resolution R = 60000 at m/z (400 mass range m/z = 150 – 2000) and dioctyl phthalate (m/z = 391.28428) as a lock mass. The high-resolution mass spectrometer was calibrated prior to measurements with a calibration mixture (Thermo Finnigan). Optical rotations were measured on a Anton Paar MCP automatic polarimeter (Sodium D-line, λ = 589 nm). LC-MS analysis was performed on a LCQ Advantage Max (Thermo Finnigan) ion-trap spectrometer (ESI+) coupled to a Surveyor HPLC system (Thermo Finnigan) equipped with a C18 column (Gemini, 4.6 mm x 50 mm, 3 µM particle size, Phenomenex) equipped with buffers A: H_2O, B: acetonitrile (MeCN) or an Agilent technologies 1260 infinity LC-MS with a 6120 Quadrupole MS system equipped with buffers A: H_2O, B: acetonitrile (MeCN) and C: 100 mM NH_4OAc. IR spectra were recorded on a Shimadzu FTIR-8300 and are reported in cm^{-1}.

3.2 Synthetic scheme for compounds 4 and 5

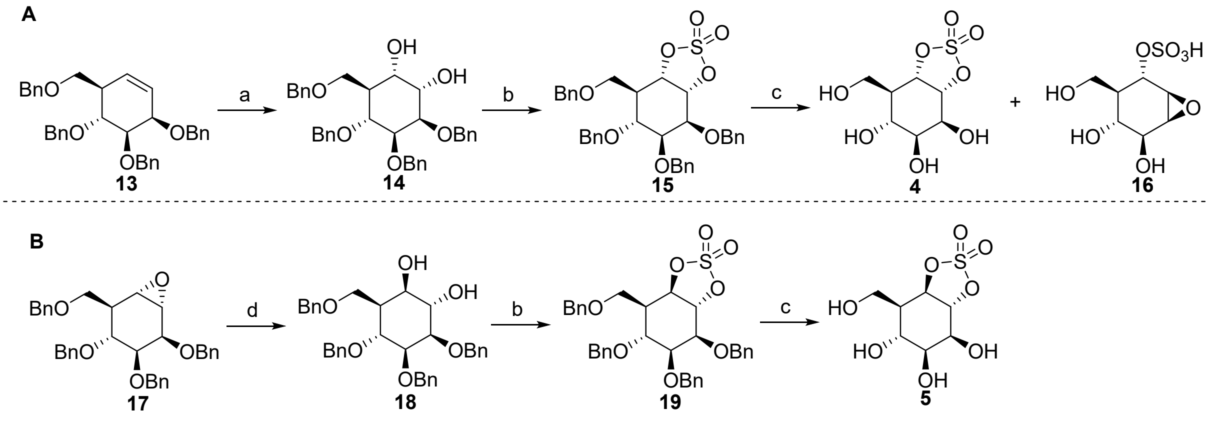

Scheme S1. Synthesis of *cis*-cyclosulfate **4** (A) and *trans*-cyclosulfate **5** (B)**.** Reagents and conditions: (a) $RuCl_3 \cdot H_2O$, $NaIO_4$, ACN, EtOAc, H_2O, 78%; (b) (i) $SOCl_2$,Et_3N, imidazole, DCM, 0 °C; (ii) $RuCl_3 \cdot H_2O$, $NaIO_4$, ACN, EtOAc, H_2O, 41% (**15**), 78% (**19**); (c) Pd/C, H_2, MeOH, 50% (**16**), 75% (**5**); (d) H_2SO_4, H_2O, THF, 80 °C, 81%.

The synthesis of **4** started with the dihydroxylation of alkene **13** which resulted exclusively in diol **14**.[11] Diol **14** was then treated with $SOCl_2$ followed by oxidation of the intermediate sulfite to obtain *cis*-cyclosulfate **15**. *Cis*-sulfate **15** was then exposed to hydrogenation conditions to obtain **4**. However, this compound could not be isoleated and proved to be unstable and convert to epoxide **16.** In contrast to the synthesis of the *cis*-manno-cyclosulfate, the synthesis of **5** was initiated by the acid hydrolysis of tetra-*O*-benzyl-α-cyclophellitol **17** which resulted in the formation of *trans*-diol **18**. The trans-diol was then treated with $SOCl_2$ followed by oxidation of the intermediate sulfite to obtain *trans*-cyclosulfate **19**, which after hydrogenation resulted in deprotected *trans*-cyclosulfate **5**, which also degraded over a week at room temperature.

3.3 Synthesis and characterization of compounds 4-6

3.3.1 Synthesis and characterization of 4

(1*S*,2*S*,3*R*,4*S*,5*R*,6*S*)-3,4,5-tris(benzyloxy)-6-((benzyloxy)methyl)cyclohexane-1,2-diol (14)

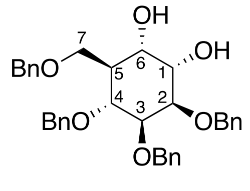

To a solution of mannose configured perbenzylated cyclohexene **13** (1.04 g, 2.0 mmol) in EtOAc/ACN (1:1, 60 mL), a solution of sodium periodate (0.64 g, 3.0 mmol, 1.5 eq.) and catalytic amount of ruthenium(III) chloride trihydrate (36 mg, 0.14 mmol, 0.07 eq.) in H_2O (16 mL) was added at 0 °C. After vigorously stirring for 1 h, the reaction mixture was quenched with 10% aqueous $Na_2S_2O_3$ (15 mL) and diluted with sat. aq. $NaHCO_3$ solution and EtOAc. The aqueous phase was separated and extracted with EtOAc (3x) and the combined organic layers were washed with brine, dried over $MgSO_4$ and concentrated *in vacuo*. Purification by column chromatography (Pentane/EtOAc from 7:3 to 6:4 gave exclusively α-diol **14** (0.746 g, 1.34 mmol, 68%) as a clear oil. ^{1}H NMR (400 MHz, $CDCl_3$): δ 7.40 – 7.24 (m, 18H, CH_{Ar}), 7.22 – 7.17 (m, 2H, CH_{Ar}), 4.89 (d, *J* = 11.0 Hz, 1H, CH*H*Ph), 4.78 (d, *J* = 12.2 Hz, 1H, CH*H*Ph), 4.69 – 4.59 (m, 3H, CH*H*Ph), 4.49 (s, 2H, CH*H*Ph), 4.45 (d, *J* = 10.9 Hz, 1H, CH*H*Ph), 4.06 – 3.91 (m, 5H, H-1, H-2, H-3, H-7A, H-7B), 3.64 (t, *J* = 9.3 Hz, 1H, H-6), 3.56 (t, *J* = 9.0 Hz, 1H, H-4), 2.60 (s, 1H, OH), 2.23 (qd, *J* = 9.4, 3.3 Hz, 1H, H-5). ^{13}C NMR (101 MHz, $CDCl_3$) δ 138.7, 138.5, 138.5, 137.4 (C_{qAr}), 128.6, 128.4, 128.1, 128.0, 127.8, 127.7, 127.6 (CH_{Ar}), 81.2 (C-1/C-2/C-3), 75.9 (C-6), 75.7 (C-1/C-2/C-

3), 75.0 (C-1/C-2/C-3), 73.6, 73.3, 72.6, (CH_2Ph), 71.8 (C-4), 71.8 (C-7), 70.0 (C-1/C-2/C-3), 42.7 (C-5). HRMS: calculated for $[C_{35}H_{38}O_6]^+$ 555.27412, found 555.27433.

(3aR,4S,5S,6R,7R,7aS)-4,5,6-tris(benzyloxy)-7-((benzyloxy)methyl)hexahydrobenzo[d][1,3,2]dioxathiole 2,2-dioxide (15)

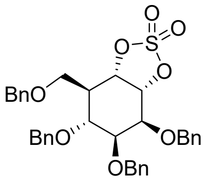

Diol **14** (0.36 g, 0.65 mmol) was dissolved in DCM (30 mL) and Et_3N (0.33 mL, 2.4 mmol, 3.7 eq.) was added at 0 °C. Next, thionyl chloride (0.15 mL, 2.1 mmol, 3.2 eq.) was slowly added over 5 min. After stirring for 1 h, additional Et_3N (90 µL, 0.65 mmol, 1 eq.) and thionyl chloride (45 µL, 0.65 mmol, 1 eq.) was added. The reaction mixture was stirred for an additional 30 min before quenching with cold H_2O and dilution with cold Et_2O. The layers were separated and the organic layer was washed with H_2O (2x), dried over $MgSO_4$, filtered and concentrated *in vacuo*. Purification by column chromatography (3.5% EtOAc in pentane) gave the sulphite diastereomeric mixture (390 mg) as an orange oil. HRMS: calculated for $[C_{35}H_{37}O_7S]^+$ 601.22545, found 601.22584. Afterwards, to a solution of the sulphite diastereomeric mixture (192 mg, 0.32 mmol) in CCl_4/MeCN (1:1, 24 mL) was added a solution of sodium periodate (0.137 g, 0.64 mmol, 2.0 eq.) and a catalytic amount of ruthenium(III) chloride trihydrate (6 mg, 22 µmol, 0.07 eq.) in H_2O (12 mL) at 0 °C. After vigorously stirring for 1.5 h, the reaction mixture was diluted with Et_2O and H_2O. The organic layer was separated, washed with brine, dried over $MgSO_4$ and concentrated *in vacuo*. Purification by column chromatography (8% EtOAc in pentane) gave title compound **15** (81 mg, 0.131 mmol, 41% over 2 steps) as a white solid. ^{1}H NMR (400 MHz, $CDCl_3$) δ 7.40 – 7.20 (m, 18H, CH_{Ar}), 7.14 (dd, *J* = 7.3, 2.2 Hz, 2H, CH_{Ar}), 5.29 (dd, *J* = 11.2, 6.4 Hz, 1H, H-6), 5.20 (t, *J* = 6.2 Hz, 1H, H-1), 4.73 (dd, *J* = 19.8, 11.8 Hz, 1H, CH*H*Ph), 4.59 (d, *J* = 4.2 Hz, 2H, CH*H*Ph), 4.59 – 4.51 (m, 1H, CH*H*Ph), 4.43 (d, *J* = 11.7 Hz, 2H, CH*H*Ph), 4.31 (d, *J* = 11.9 Hz, 1H, CH*H*Ph), 4.22 (d, *J* = 11.5 Hz, 1H, CH*H*Ph), 4.11 (d, *J* = 1.6 Hz, 1H, H-2), 3.82 – 3.78 (m, 2H, H-3, H-4), 3.58 (dd, *J* = 9.6, 2.8 Hz, 1H, H-7B), 3.52 (dd, *J* = 9.6, 2.7 Hz, 1H, H-7A), 2.21 (ddt, *J* = 11.1, 8.2, 2.8 Hz, 1H, H-5) ^{13}C NMR (101 MHz, $CDCl_3$) δ 138.0, 137.6, 137.5, 137.4 (C_{qAr}), 128.7, 128.6, 128.5, 128.2, 128.1, 128.0, 127.9, 127.7 (CH_{Ar}), 83.6 (C-1) , 80.0 (C-6), 76.5 (C-2), 76.2 (C-3), 74.2 (C-4), 73.2, 72.9, 72.8, 72.6 (CH_2Ph), 64.8 (C-7), 42.5 (C-5). HRMS: calculated for $[C_{35}H_{36}O_8SNa]^+$ 639.20231, found 639.20238.

(3a*R*,4*S*,5*S*,6*R*,7*R*,7*aS*)-4,5,6-trihydroxy-7-(hydroxymethyl)hexahydrobenzo[*d*][1,3,2]dioxathiole 2,2-dioxide (16)

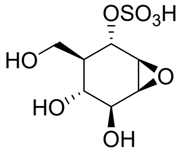

Benzylated sulphate **15** (70 mg, 0.11 mmol) was dissolved in MeOH (10 mL). The reaction mixture was purged with nitrogen gas and 10% palladium on carbon (15 mg) was added. Afterwards, the reaction vessel was purged with hydrogen and vigorously stirred overnight. The palladium catalyst was then filtered off followed by concentration *in vacuo*. Purification by column chromatography (10% MeOH in DCM → 20% MeOH in DCM) yielded compound **16** (14 mg, 55 µmol, 50 %) as a clear oil. ^{1}H NMR (500 MHz, MeOD) δ 4.20 (d, *J* = 2.0 Hz, 1H, H-1), 4.15 (d, *J* = 5.2 Hz, 1H, H-6), 4.08 (d, *J* = 5.3 Hz, 1H, H-4), 4.04 – 3.97 (m, 1H, H-2), 3.79 – 3.73 (m, 1H, H-3), 3.59 (dd, *J* = 10.6, 6.3 Hz, 1H, H-7B), 3.51 – 3.43 (m, 1H, H-7A), 2.25 – 2.20 (m, 1H, H-5). ^{13}C NMR (126 MHz, MeOD) δ 88.3 (C-1), 83.2 (C-6), 82.6 (C-3), 76.2 (C-2), 74.3 (C-4), 64.1 (C-7), 45.7 (C-5).

3.3.2 Synthesis and characterization of 5

(1*R*,2*S*,3*R*,4*S*,5*R*,6*S*)-3,4,5-tris(benzyloxy)-6-((benzyloxy)methyl)cyclohexane-1,2-diol (18)

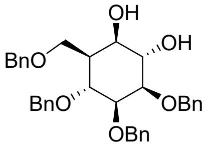

Fully benzylated β-mannose-configured cyclophellitol **17** (1 g, 1.86 mmol) was dissolved in THF (10 mL) and 3 M aq. H_2SO_4 (20 mL) was added and the resultant mixture was stirred at 80 ºC for 18 h. By TLC one main new spot can be observed. The reaction mixture was then allowed to cool to rt and the residue was dissolved in Et_2O (50 mL) and quenched with $NaHCO_3$ and extracted with Et_2O. The combined organic layers were dried over $MgSO_4$ and concentrated in *vacuo*. Purification by silica gel column chromatography (pentane /EtOAc 70:30 → 60:40) yielded **18** (0.84 g, 1.51 mmol, 81%). ^{1}H NMR (400 MHz, $CDCl_3$) δ 7.35 – 7.17 (m, 20H, CH_{Ar}), 4.63 – 4.37 (m, 8H, CH*H*Ph), 4.03 – 3.94 (m, 2H, H-1, H-6), 3.92 – 3.79 (m, 3H, H-3, H-4, H-7B), 3.74 – 3.66 (m, 2H, H-2, H-7B), 3.45 (s, 1H, OH), 2.85 (s, 1H, OH), 2.62 (q, *J* = 6.7, 5.9 Hz, 1H, H-5). ^{13}C NMR (101 MHz, $CDCl_3$) δ 138.3, 138.2, 138.1 (C_{qAr}), 128.5, 128.4, 128.4, 128.4, 128.0, 127.9, 127.9, 127.8, 127.7, 127.7 (CH_{Ar}), 79.8 (C-2), 74.8 (C-3/4), 73.2, 72.9, 72.8 (CH_2Ph), 71.6 (C-1), 70.7 (C-6), 67.9 (C-7), 42.7 (C-5).

(3*aR*,4*S*,5*S*,6*R*,7*R*,7*aR*)-4,5,6-tris(benzyloxy)-7-((benzyloxy)methyl)hexahydrobenzo[*d*][1,3,2]dioxathiole 2,2-dioxide (19)

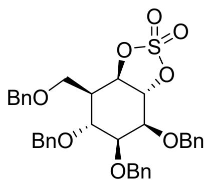

To a solution of the diol **18** (96 mg, 0.17 mmol), Et_3N (0.12 mL, 0.87 mmol, 5 eq) and imidazole (59 mg, 0.87 mmol, 5 eq) in DCM (10 mL) at 0 ºC was added $SOCl_2$ (0.11 mL, 1.56 mmol) over 5 min and stirred for 2 h. The reaction mixture was diluted with cold Et_2O (20 mL) and washed with cold water (2 x 20 mL) and brine (30 mL). The organic layer was dried over $MgSO_4$ and filtered. The filtrate was concentrated and the residual triethylamine was removed under high vacuum (ca. 1 h). The resulting oil was dissolved in MeCN (4 mL) and CCl_4 (4 mL), and the solution cooled in an ice-bath. A solution of catalytic amount of $RuCl_3{\cdot}3H_2O$ and $NaIO_4$, (74 mg, 0.35 mmol) in H_2O was added and the reaction was stirred at rt overnight. EA (20 mL) and H2O (20 mL) were added and the two layers were separated. The aqueous layer was extracted with Et_2O (2 x25 mL) and the combined organic extracts were washed with brine (2 x 25 mL), dried over $MgSO_4$ filtered and concentrated *in vacuo*. Purification by column chromatography (8% EtOAc in pentane) gave title compound **19** (83 mg, 0.13 mmol, 78% over 2 steps). ^{1}H NMR (400 MHz, $CDCl_3$) δ 7.38 – 7.22 (m, 17H, CH_{Ar}), 7.16 (ddd, *J* = 11.1, 6.6, 2.1 Hz, 3H, CH_{Ar}), 5.18 (t, *J* = 10.4 Hz, 1H, H-1), 5.00 (dd, *J* = 10.8, 5.9 Hz, 1H, H-6), 4.72 (d, *J* = 11.8 Hz, 1H, CH*H*Ph), 4.58 (d, *J* = 11.9 Hz, 1H, CH*H*Ph), 4.55 – 4.35 (m, 5H, 5x CH*H*Ph), 4.38 – 4.28 (m, 1H, CH*H*Ph), 4.11 (t, *J* = 2.7 Hz, 1H, H-4), 4.02 (dd, *J* = 10.0, 3.3 Hz, 1H, H-2), 3.89 (t, *J* = 3.3 Hz, 1H, H-3), 3.82 (t, *J* = 9.7 Hz, 1H, H-7B), 3.71 (dd, *J* = 9.3, 5.2 Hz, 1H, H-7A), 2.94 (dtd, *J* = 10.7, 5.6, 1.9 Hz, 1H, H-5). ^{13}C NMR (101 MHz, $CDCl_3$) δ 137.9, 137.5, 137.4, 137.0 (4x C_{qAr}), 128.7, 128.7, 128.6, 128.6, 128.6, 128.4, 128.2, 128.1, 128.0, 127.9, 127.8, 127.7 (CH_{Ar}), 84.6 (C-1), 84.0 (C-6), 77.5 (C-3), 76.3 (C-2), 74.7 (C-4), 74.1, 73.4, 72.8, 72.5 (4x CH_2Ph), 65.0 (C-7), 41.1 (C-5).

(3*aR*,4*S*,5*S*,6*R*,7*R*,7*aR*)-4,5,6-trihydroxy-7-(hydroxymethyl)hexahydrobenzo[d][1,3,2]dioxathiole 2,2-dioxide (5)

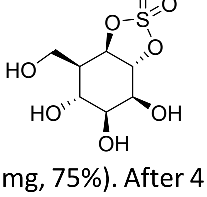

20% Palladium hydroxide on carbon (13 mg, 0.018 mmol) was added to a solution of **19** (28 mg, 0.045 mmol) in anhydrous MeOH (5 mL) under Argon atmosphere. The mixture stirred under hydrogen atmosphere for 18 h. Then, filtered through a celite plug and the solvent was evaporated under reduced pressure. The crude was purified by silica flash column chromatography (DCM → DCM/MeOH 9:1) to afford **5** (8.78 mg, 75%). After 4 months in the fridge around 40% degradation is observed. ^{1}H NMR (400 MHz, MeOD) δ 5.16 – 5.03 (m, 2H, H-1, H-6), 4.28 (dd, *J* = 3.1, 2.1 Hz, 1H, H-4), 4.18 – 4.11 (m, 1H, H-2), 4.02 (td, *J* = 3.3, 0.9 Hz, 1H, H-3), 3.98 (dd, *J* = 11.3, 7.2 Hz, 1H, H-7A), 3.84 (dd, *J* = 11.4, 4.8 Hz, 1H, H-7B), 2.63

(dtdd, *J* = 6.9, 4.8, 2.1, 0.9 Hz, 1H, H-5). ^{13}C NMR (101 MHz, MeOD) δ 85.6 (C-1/C-6), 84.7 (C-1/C-6), 74.1 (C-3), 71.0 (C-4), 68.5 (C-2), 56.9 (C-7), 45.9 (C-5).

3.3.3 Synthesis and characterization of 6

(1*R*,2*S*,3*R*,4*R*,5*S*,6*R*)-2-azido-4,5,6-tris(benzyloxy)-3-((benzyloxy)met-hyl)-cyclohexan-1-ol (8) and (1*R*,2*S*,3*R*,4*R*,5*S*,6*R*)-2-azido-4,5,6-tris(benzyloxy)-3-((benzyloxy)methyl)-cyclohexan-1-ol (9) Fully benzylated β-mannose-configured cyclophellitol **7** (637 mg, 1.19 mmol) was dissolved in anhydrous DMF (30 mL). $LiClO_4$ (1.66 g, 15.6 mmol, 13.0 eq) and NaN_3 (1.01 g, 15.6 mmol, 13.0 eq) were added and the reaction was stirred under N_2 atmosphere overnight at 100 °C. The reaction mixture as cooled to rt and quenched with H_2O. The product was extracted with EtOAc (3x), washed with brine (2x) dried over MgSO4 and concentrated *in vacuo.* Purification by silica column chromatography (pentane /EtOAc 75:25 → 65:35) yielded a 1:1 mixture of two diastereomers: **8** (0.40 g, 0.69 mmol, 58%) and 1-hydroxy-6-azido **9** (93.5 mg, 0.161 mmol, 14%).

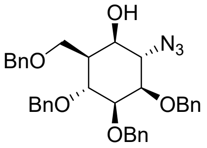

Azide (8): $[\alpha]_D^{20}$ = -19.4 (*c* = 1, $CHCl_3$). IR (neat, cm^{-1}) 3390, 2869, 2109, 1497, 1454, 1265, 1066, 1027, 912, 733, 695. ^{1}H NMR (400 MHz, $CDCl_3$) *δ* 7.39 – 7.14 (m, 20H, CH Ar), 4.71 – 4.39 (m, 8H, 4CH_2Ph), 3.90 (dt, *J* = 14.6, 8.7 Hz, 3H, H7a), 3.75 (t, *J* = 4.7 Hz, 1H), 3.72 (t, *J* = 3.9 Hz, 1H), 3.67 (dd, *J* = 8.1, 2.9 Hz, 1H), 3.62 (dd, *J* = 9.5, 5.5 Hz, 1H, H-7b), 3.45 (s, 1H, OH), 2.57 (t, *J* = 6.0 Hz, 1H, H-5).^{13}C NMR (101 MHz, $CDCl_3$) *δ* 138.1, 138.0, 137.8 (4C_q Ar), 128.6 - 127.8 (20CH Ar), 78.7 (CH), 75.1 (CH), 73.4, 73.3, 73.2 (4CH_2Ph), 70.7 (CH), 68.1 (C-7), 42.8 (C-5) HRMS: Calcd. for $[C_{35}H_{37}N_3O_5{\cdot}NH_4]^+$ m/z 597.30730; found m/z 597.30715. HRMS: Calcd. for $[C_{35}H_{37}N_3O_5{\cdot}Na]^+$ m/z 602.26225; found m/z 602.26254.

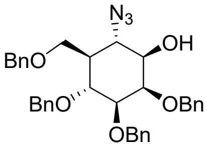

Azide (9): $[\alpha]_D^{20}$ = -35.6 (*c* = 1, $CHCl_3$). IR (neat, cm^{-1}) 2868, 2106, 1454, 1264, 1089, 1027, 731, 697. ^{1}H NMR (400 MHz, $CDCl_3$) *δ* 7.42 – 7.15 (m, 20H, CH Ar), 5.16 (d, *J* = 11.7 Hz, 1H, CH*H*Ph), 4.90 (d, *J* = 10.7 Hz, 1H, CH*H*Ph), 4.75 (dd, 2H, CH_2Ph), 4.67 (d, *J* = 11.7, 1.5 Hz, 1H, CH*H*Ph), 4.56 – 4.49 (m, 2H, CH_2Ph), 4.46 (d, *J* = 12.1 Hz, 1H, CH*H*Ph), 4.10 (t, *J* = 10.3 Hz, 1H), 4.06 (t, *J* = 2.6 Hz, 1H), 3.88 – 3.76 (m, 2H, H-7a), 3.63 (dd, *J* = 9.2, 2.3 Hz, 1H, H-7b), 3.47 – 3.37 (m, 2H), 1.34 (td, *J* = 11.2, 2.0 Hz, 1H, H-5). ^{13}C NMR (101 MHz, $CDCl_3$) *δ* 138.6, 138.6, 138.5, 138.3 (4C_q Ar), 128.7 - 127.7 (20CH Ar), 84.2 (CH), 77.8 (CH), 75.8 (CH), 75.7, 74.8 (2CH_2Ph), 74.2 (CH), 73.2, 73.2 (2CH_2Ph), 65.1 (C-7), 62.4 (CH), 45.0 (C-5). HRMS: Calcd. for $[C_{35}H_{37}N_3O_5{\cdot}NH_4]^+$ m/z 597.30750; found m/z 597.30715. HRMS: Calcd. for $[C_{35}H_{37}N_3O_5{\cdot}Na]^+$ m/z 602.26225; found m/z 602.26286.

(1*R*,2*S*,3*R*,4*S*,5*R*,6*S*)-2-amino-3,4,5-tris(benzyloxy)-6-((benzyloxy)met-hyl)-cyclohexan-1-ol (10)

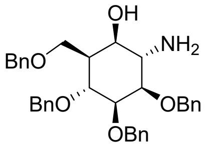

Azide **8** (400 mg, 0.69 mmol, 58%) was dissolved in anhydrous THF (45 mL) and purged with N_2 for 10 minutes. PtO_2 (47 mg, 0.21 mmol, 0.3 eq) was added and the mixture was bubbled with N_2 for another 10 minutes. Subsequently, the reaction was stirred under H_2 atmosphere overnight at rt. After complete conversion, the catalyst was removed by filtration over Celite and the filtrate was concentrated *in vacuo.* Purification by silica column chromatography (pentane /EtOAc 75:25 → 65:35) yielded amine **10** (0.33 g, 0.60 mmol, 86%). $[\alpha]_D^{20}$ = 2.9 (*c* = 1, $CHCl_3$). IR (neat, cm^{-1}) 2923, 2866, 1497, 1454, 1066, 1027, 733, 695. ^{1}H NMR (400 MHz, $CDCl_3$) *δ* 7.35 – 7.13 (m, 20H, CH Ar), 4.59 – 4.36 (m, 8H), 3.98 – 3.85 (m, 3H, H-7a), 3.76 (t, *J* = 3.6 Hz, 1H), 3.66 (t, 1H, H-7b), 3.59 (dd, *J* = 9.4, 3.0 Hz, 1H), 3.32 – 3.11 (m, 3H), 2.70 – 2.60 (m, 1H, CH-5). ^{13}C NMR (101 MHz, $CDCl_3$) *δ* 138.3, 138.3, 138.1 (4C_q Ar), 128.5 - 127.7 (20CH Ar), 80.2 (CH), 75.3 (CH), 74.8 (CH), 73.2, 72.7, 72.3, 71.3 (4CH_2Ph), 71.3 (CH), 68.2 (C-7), 52.9 (CH), 42.6 (C-5). HRMS: Calcd. for $[C_{35}H_{39}NO_5{\cdot}H]^+$ m/z 554.28999; found m/z 554.29010.

Benzyl ((1*S*,2*R*,3*S*,4*R*,5*S*,6*R*)-2,3,4-tris(benzyloxy)-5-((benzyloxy)met-hyl)-6-hydroxycyclohexyl)carbamate (11): Amine **10** (0.15 g, 0.27 mmol) was dissolved in a solution of dioxane and water (1.5:1). K_2CO_3 (12 mg, 85 µmol, 1 eq) was added and the mixture was stirred for 10 minutes. Benzyl chloroformate (15 µL, 0.18 mmol, 1.2 eq) was added and the reaction was vigorously stirred for 1 h. After complete conversion the mixture was diluted with DCM, washed with water (1x) and brine (1x), dried over $MgSO_4$ and concentrated *in vacuo.* Purification by silica column chromatography (pentane /EtOAc 80:20 → 65:35) yielded Cbz-protected amine **11** (150 mg, 0.2 mmol, 74%). $[\alpha]_D^{20}$ = +8.67 (*c* = 0.3, $CHCl_3$). IR (neat, cm^{-1}): 2865, 1709, 1454, 1093, 736, 697. ^1H NMR (400 MHz, $CDCl_3$) δ 7.40 – 7.16 (m, 25H, CH Ar), 5.14 – 5.05 (m, 2H, CH_2PhCbz), 4.64 (d, *J* = 11.3 Hz, 3H), 4.58 – 4.47 (m, 5H), 4.42 (d, *J* = 11.9 Hz, 1H), 4.17 (dd, *J* = 6.7 Hz, 1H), 4.01 (dd, *J* = 11.0, 5.2 Hz, 1H), 3.96 – 3.81 (m, 2H, CH-7a), 3.79 – 3.73 (m, 1H, CH-7b), 3.70 (dd, *J* = 6.6, 2.9 Hz, 1H), 2.28 (m, 1H). ^{13}C NMR (101 MHz, $CDCl_3$) δ 138.4, 138.3, 138.2, 137.7, 136.4 ($5C_q$ Ar), 128.7 - 127.7 (25CH Ar), 78.2 (CH), 77.6 (CH), 74.8 (CH), 73.4, 73.0, 72.7 ($4CH_2Ph$), 70.6 (CH), 67.6 (C-7), 67.2 (CH_2PhCbz), 52.7 (CH), 44.1 (C-5). HRMS: Calcd. for $[C_{43}H_{45}NO_7{\cdot}H]^+$ m/z 688.32671; found m/z 688.32688.

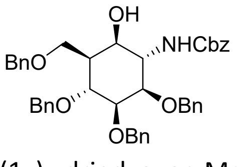

Benzyl (3a*R*,4*R*,5*S*,6*R*,7*R*,7a*R*) -4,5,6-tris(benzyloxy)-7-((benzyloxy)-methyl) - hexahydro-3H-benzo[*d*][1,2,3]oxathiazole-3-carboxylate 2,2-dioxide (12): Cbz-protected amine **11** (0.15 g, 0.17 mmol) was dissolved in dry DCM (3 mL). Imidazole (64 mg, 0.94 mmol, 5.5 eq) and Et_3N (249 µL, 1.79 mmol, 10.5 eq) were added at 0 °C. $SOCl_2$ (123 µL, 0.202 g, 1.70 mmol) was added after 5 minutes at neutral pH. After 1 h the mixture was diluted in cold Et_2O, washed with brine (1x), dried over $MgSO_4$ and concentrated under high vacuum for 1 h. The crude material was dissolved in EtOAc (2.5 mL), CAN (2.5 mL) and H_2O (2.5 ml) and the mixture was cooled to 0 °C. $NaIO_4$ (91 mg, 0.43 mmol, 2.5 eq) was added and subsequently $RuCl_3$ (8.8 mg, 43 µmol, 0.25 eq) and the mixture was stirred for 3 h. The mixture was diluted with EtOAc, washed with sat. aq. $Na_2S_2O_3$, H_2O and brine, dried over $MgSO_4$ and concentrated *in vacuo.* Purification by silica column chromatography (pentane /EtOAc 88:12 → 92:8) yielded the protected cyclosulfamidate **12** (59 mg, 78 µmol, 46%). ^1H NMR (400 MHz, $CDCl_3$) δ 7.44 – 7.04 (m, 25H), 5.34 (d, *J* = 12.1 Hz, 1H, CH*H*PhCbz), 5.20 (d, *J* = 12.1 Hz, 1H, CH*H*PhCbz), 4.85 (dd, *J* = 11.5, 5.2 Hz, 1H), 4.79 – 4.71 (m, 1H), 4.61 (d, *J* = 12.1 Hz, 1H, CH*H*Ph), 4.49 (d, *J* = 15.9 Hz, 1H, CH*H*Ph), 4.44 (d, 1H, CH*H*Ph), 4.40 (dd, *J* = 7.0, 5.0 Hz, 3H, 3x CH*H*Ph), 4.29 (t, *J* = 12.0 Hz, 2H, CH_2Ph), 4.07 (t, *J* = 2.5 Hz, 1H), 3.86 (t, *J* = 9.6 Hz, 1H, H-7a), 3.81 – 3.77 (m, 2H), 3.67 (dd, *J* = 9.2, 4.9 Hz, 1H, H-7b), 2.90 (dtd, *J* = 10.1, 5.1, 1.9 Hz, 1H, H-5). ^{13}C NMR (101 MHz, $CDCl_3$) δ 151.9 (C=O Cbz), 138.0, 137.8, 137.7, 137.3, 134.6 (5Cq Ar), 128.7 - 127.7 (25CH Ar), 79.5 (CH), 78.4 (CH), 76.4 (CH), 74.5 (CH), 73.7, 73.4, 72.5, 72.4 ($4CH_2Ph$), 69.9 (CH_2PhCbz), 65.2 (C-7), 59.4 (CH), 40.9 (C-5).

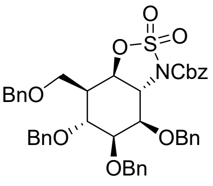

(3a*R*,4*R*,5*S*,6*R*,7*R*,7a*R*)-4,5,6-trihydroxy-7-(hydroxymethyl)hexahydro-3H-benzo[d][1,2,3]oxathiazole 2,2-dioxide (6): The protected cyclosulfamidate **12** (27 mg, 36 µmol) was dissolved in MeOH (7.8 mL) and purged with N_2. Pd/C (10 wt%, 34 mg, 31 µmol, 0.4 eq) was added and the mixture was again purged with N_2. Subsequently, the mixture was put under H_2 atmosphere and stirred overnight at rt. The reaction mixture was filtered over a celite plug and concentrated *in vacuo.* Purification by silica column chromatography (DCM /MeOH 95:5 → 85:15) yielded cyclosulfamidate **6** (5.1 mg, 20 µmol, 75%). ^1H NMR (400 MHz, MeOD) δ 4.76 (dd, *J* = 10.6, 5.8 Hz, 1H, H-6), 4.30 (t, *J* = 2.5 Hz, 1H, H-3), 4.05 (d, *J* = 9.7 Hz, 1H, H-4), 4.01 (d, *J* = 3.5 Hz, 1H, H-7a), 4.00 – 3.96 (m, 1H, H-1), 3.96 – 3.94 (m, 1H, H-2), 3.83 (dd, *J* = 11.2, 4.5 Hz, 1H, H-7b), 2.50 (qd, *J* = 6.9, 5.9, 1.9 Hz, 1H, CH-5). ^{13}C NMR (101 MHz, MeOD) δ 86.6 (C-6), 74.8 (C-2), 72.4 (C-3), 70.4 (C-1), 60.6 (C-4), 58.5 (C-7), 46.8 (C-5)

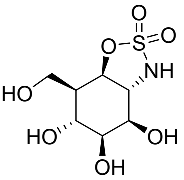

4. NMR spectra

^{1}H-NMR and ^{13}C-NMR spectra of **8** in $CDCl_3$

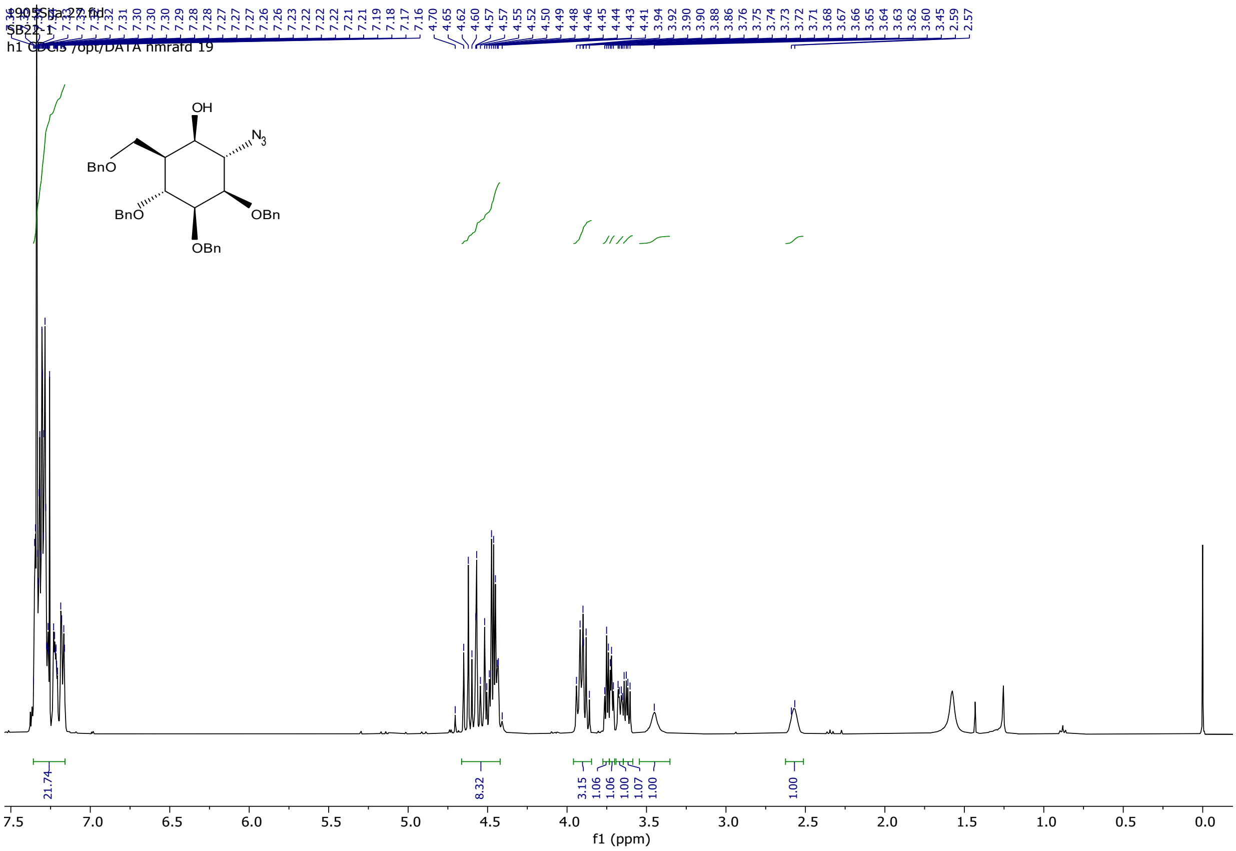

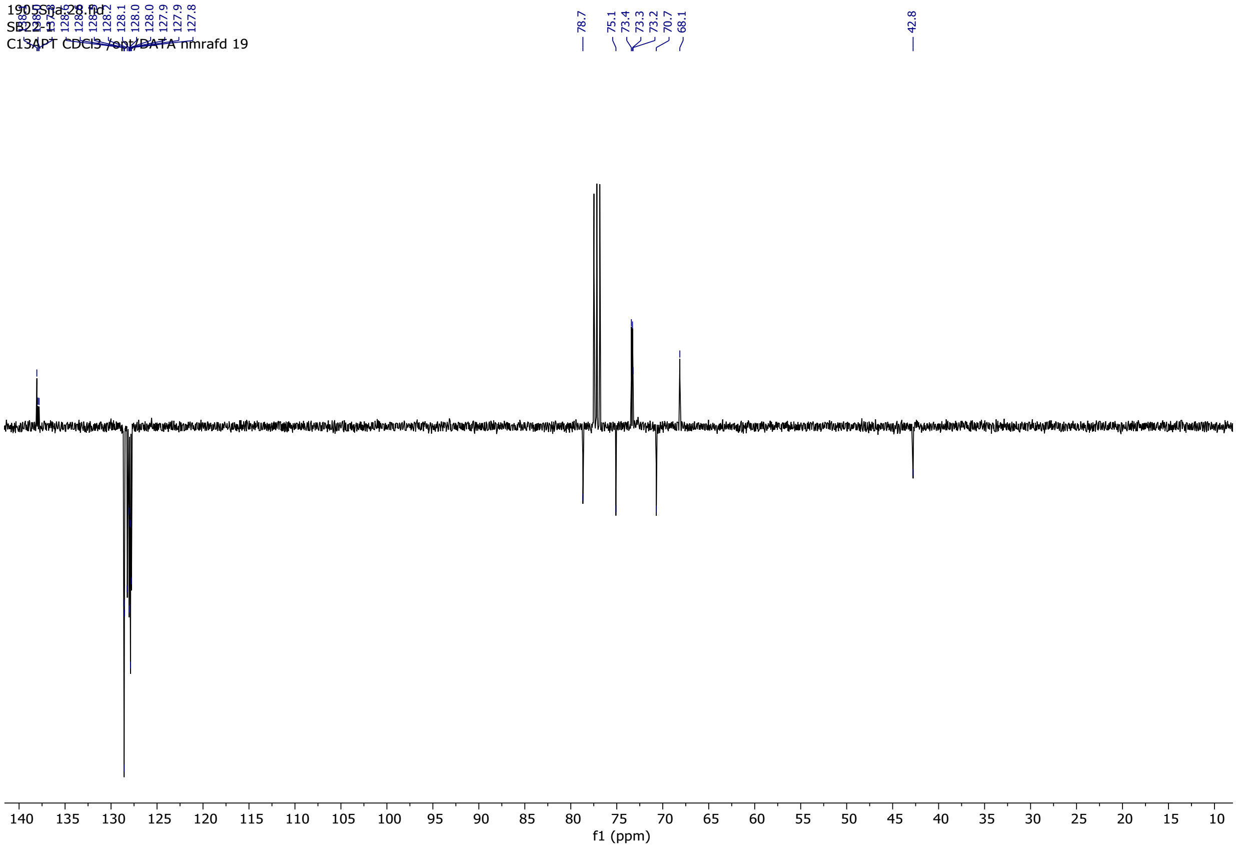

^{1}H-NMR and ^{13}C-NMR spectra of **9** in $CDCl_3$

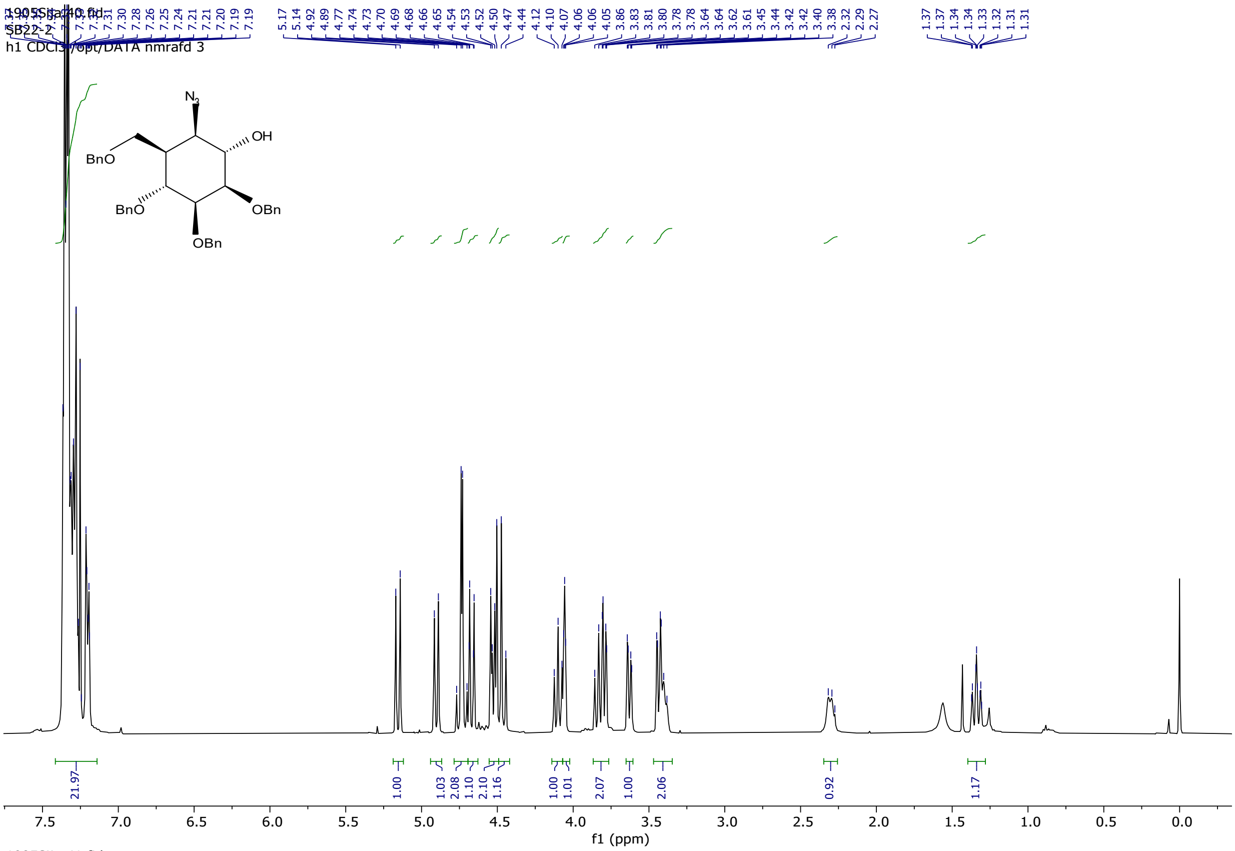

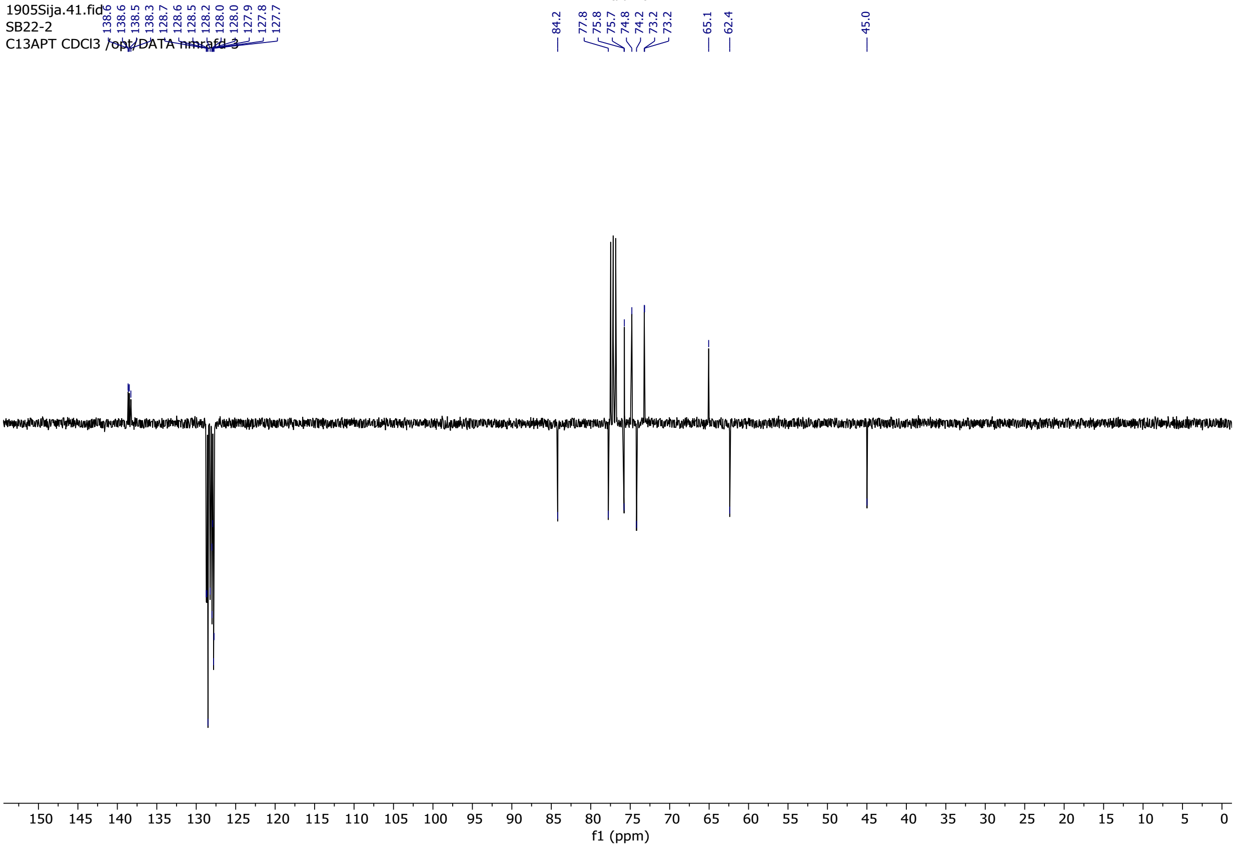

^{1}H-NMR and ^{13}C-NMR spectra of **10** in $CDCl_3$

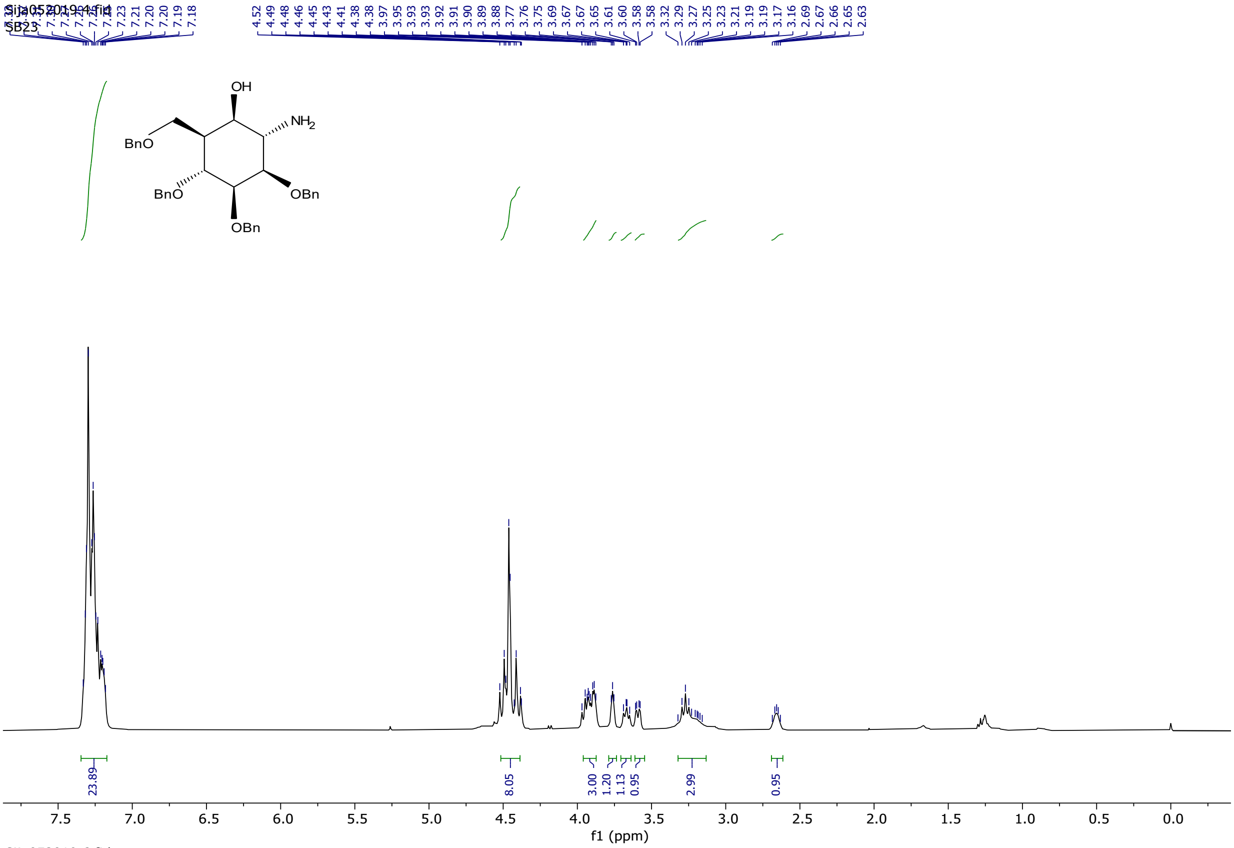

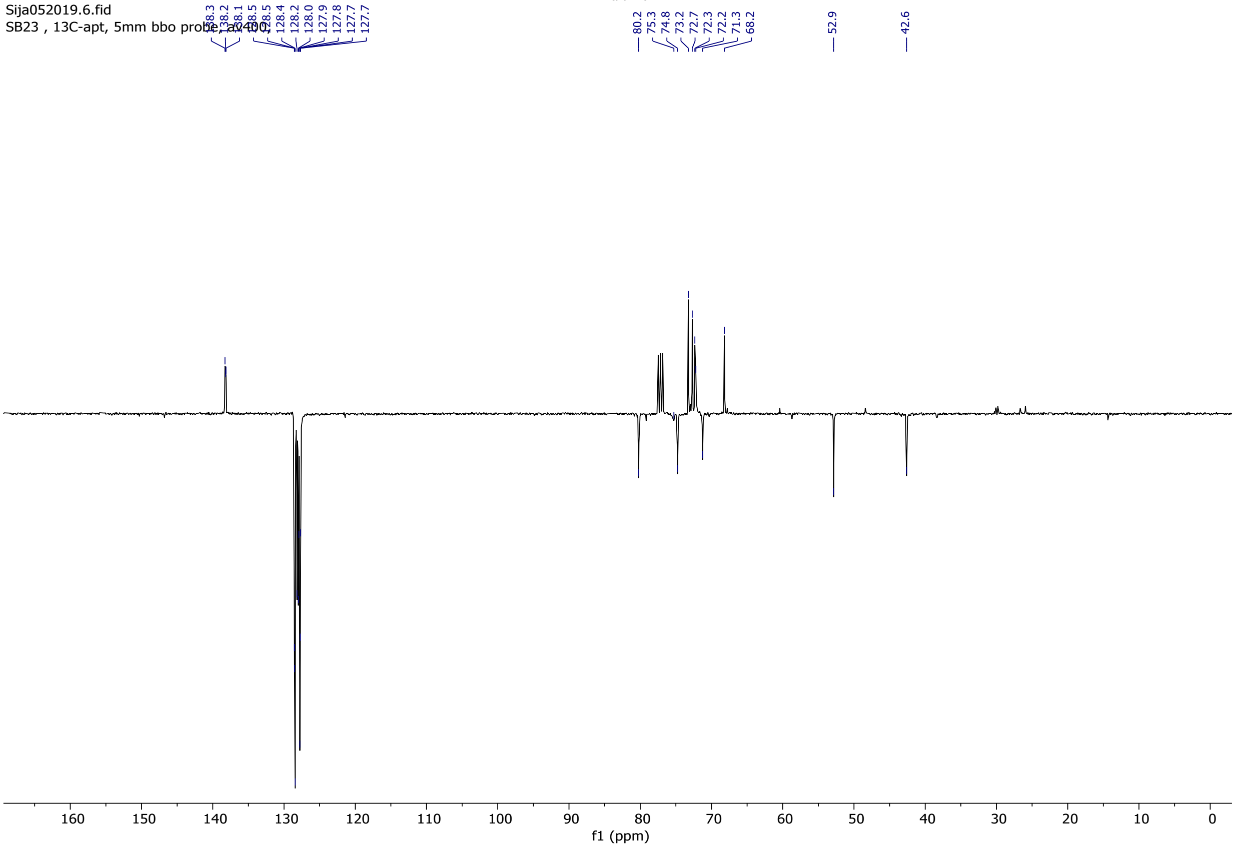

^{1}H-NMR and ^{13}C-NMR spectra of **11** in $CDCl_3$

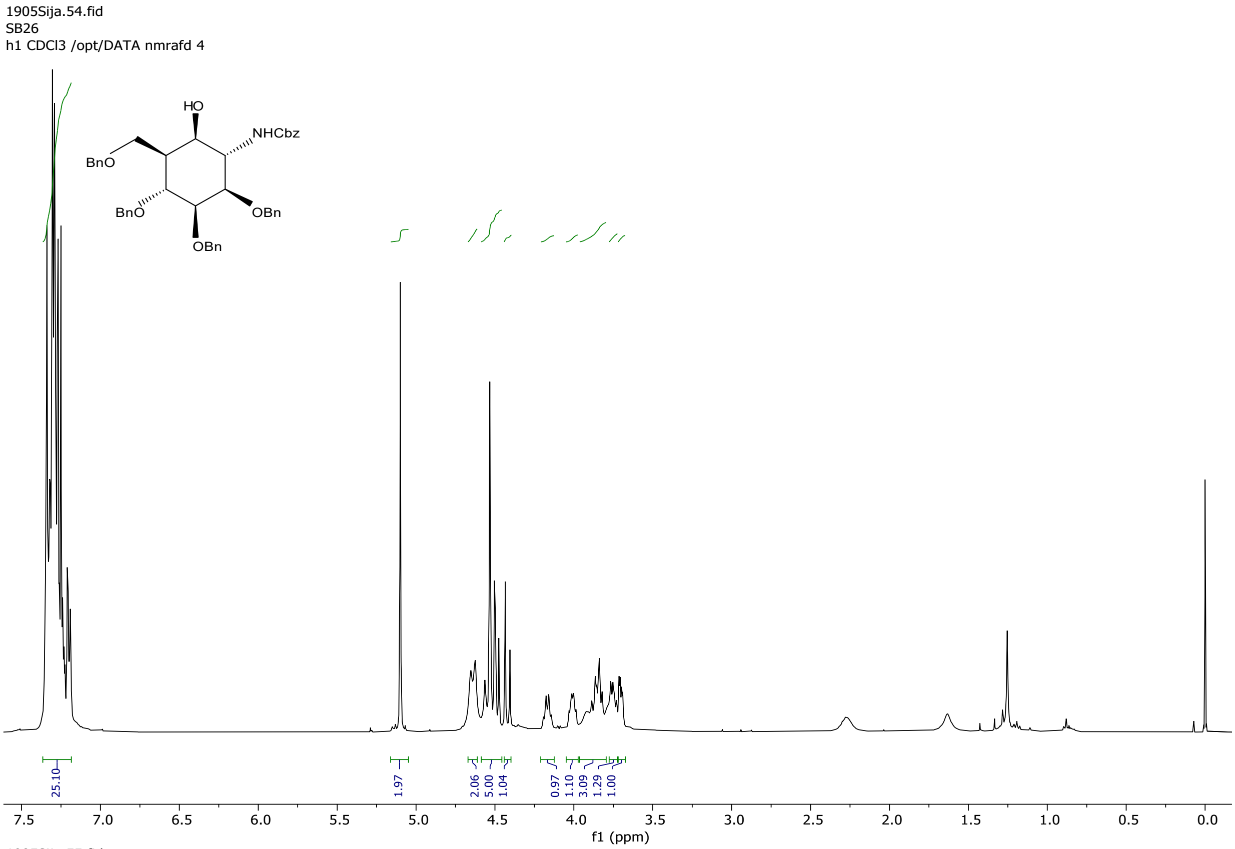

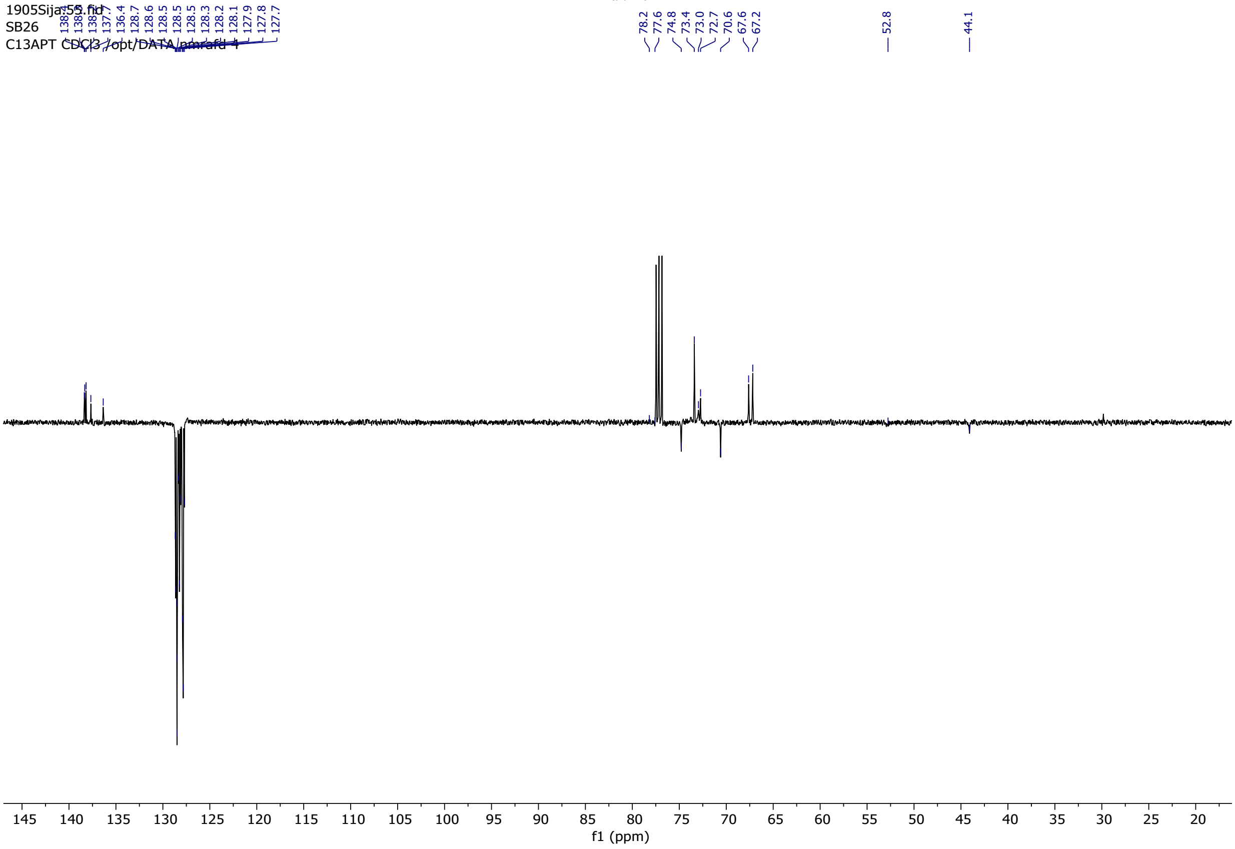

^{1}H-NMR and ^{13}C-NMR spectra of **12** in $CDCl_3$

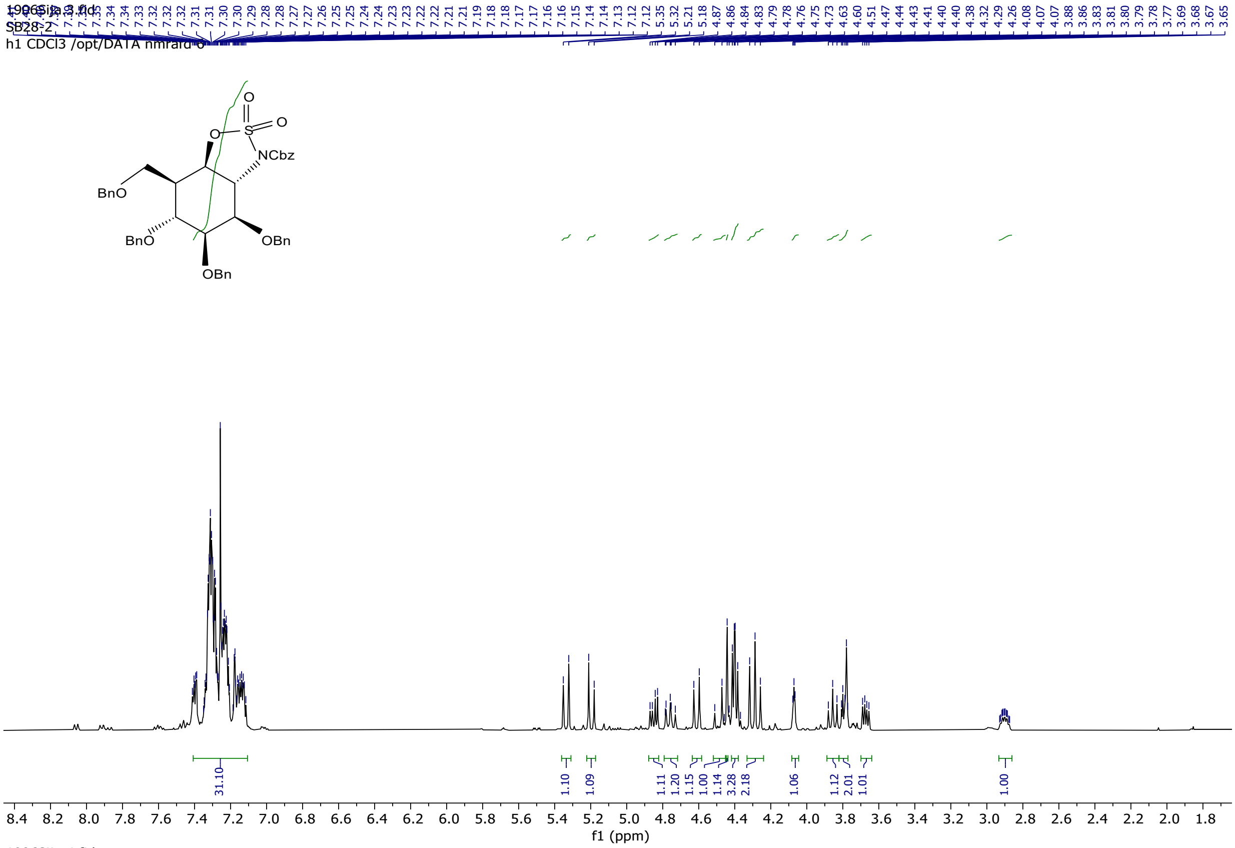

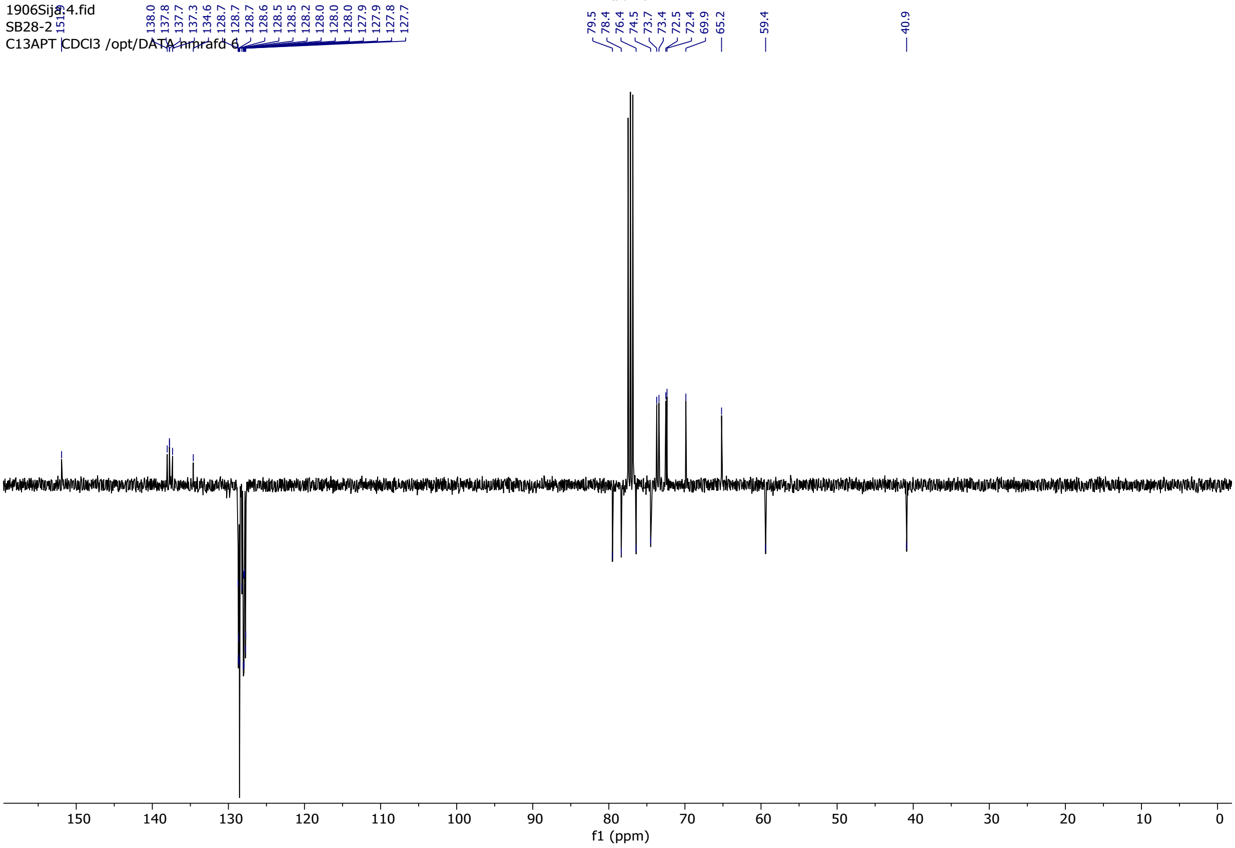

^{1}H-NMR and ^{13}C-NMR spectra of **14** in $CDCl_3$

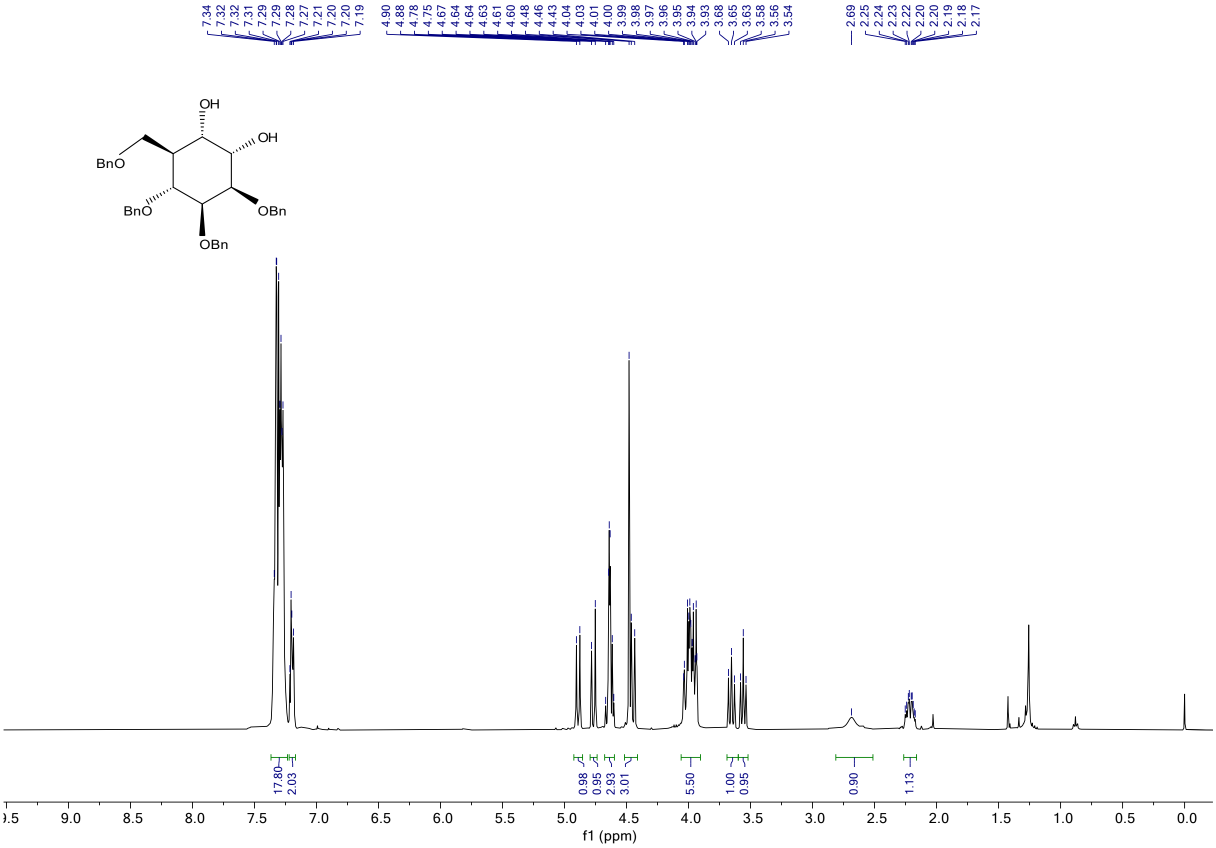

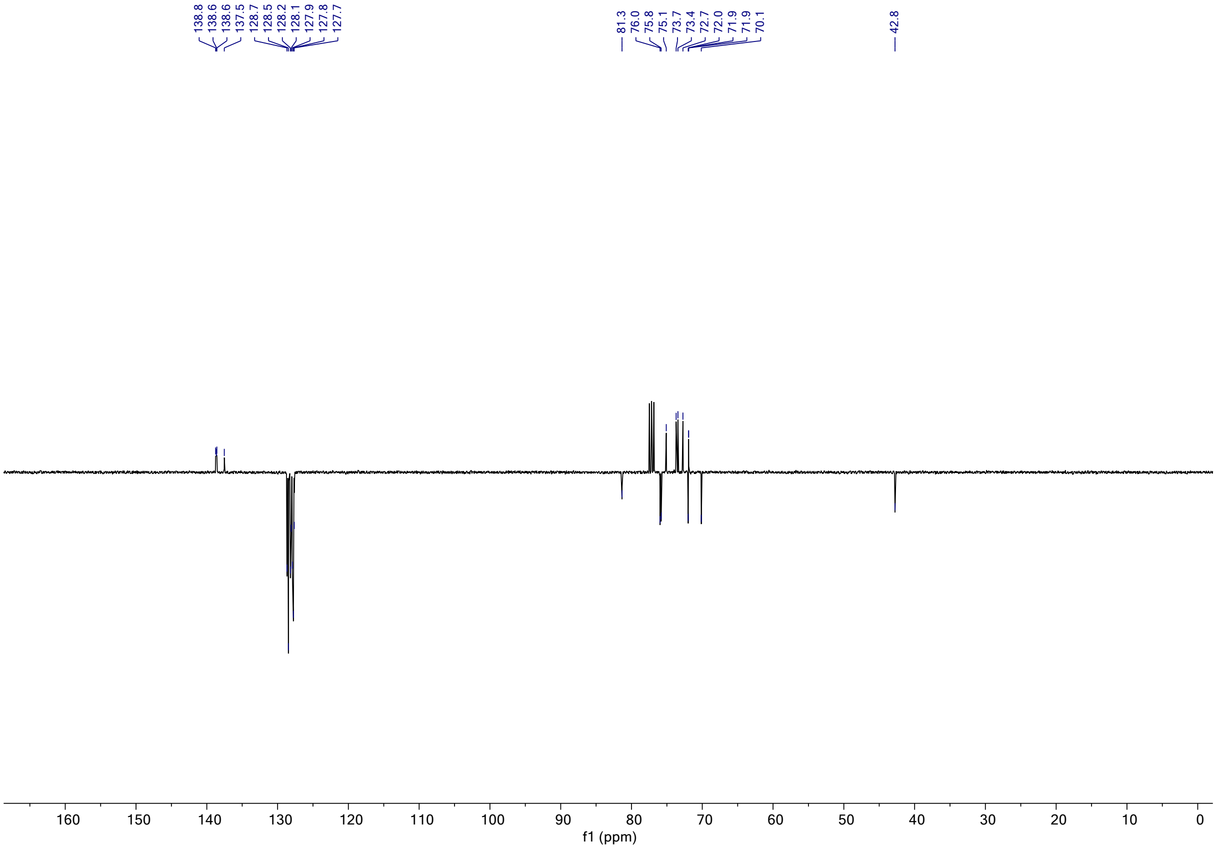

^{1}H-NMR and ^{13}C-NMR spectra of **15** in $CDCl_3$

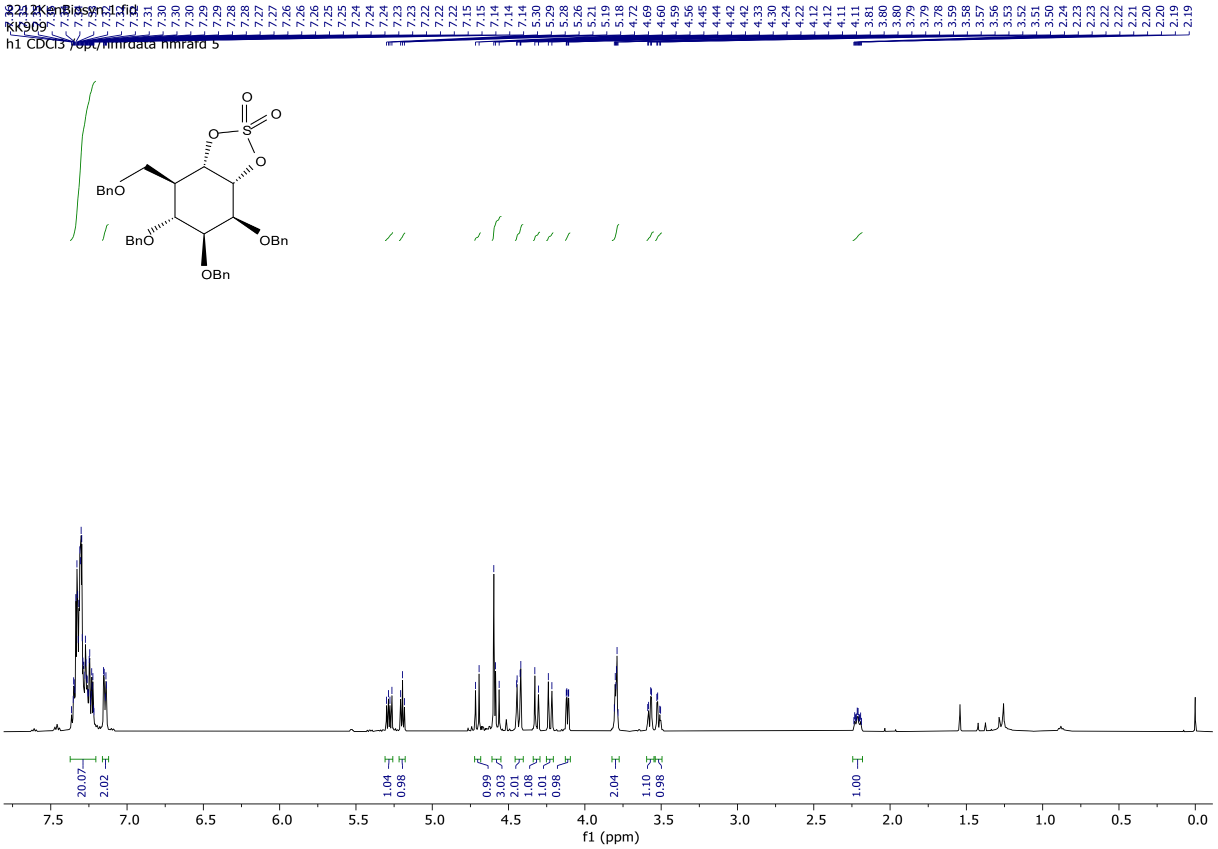

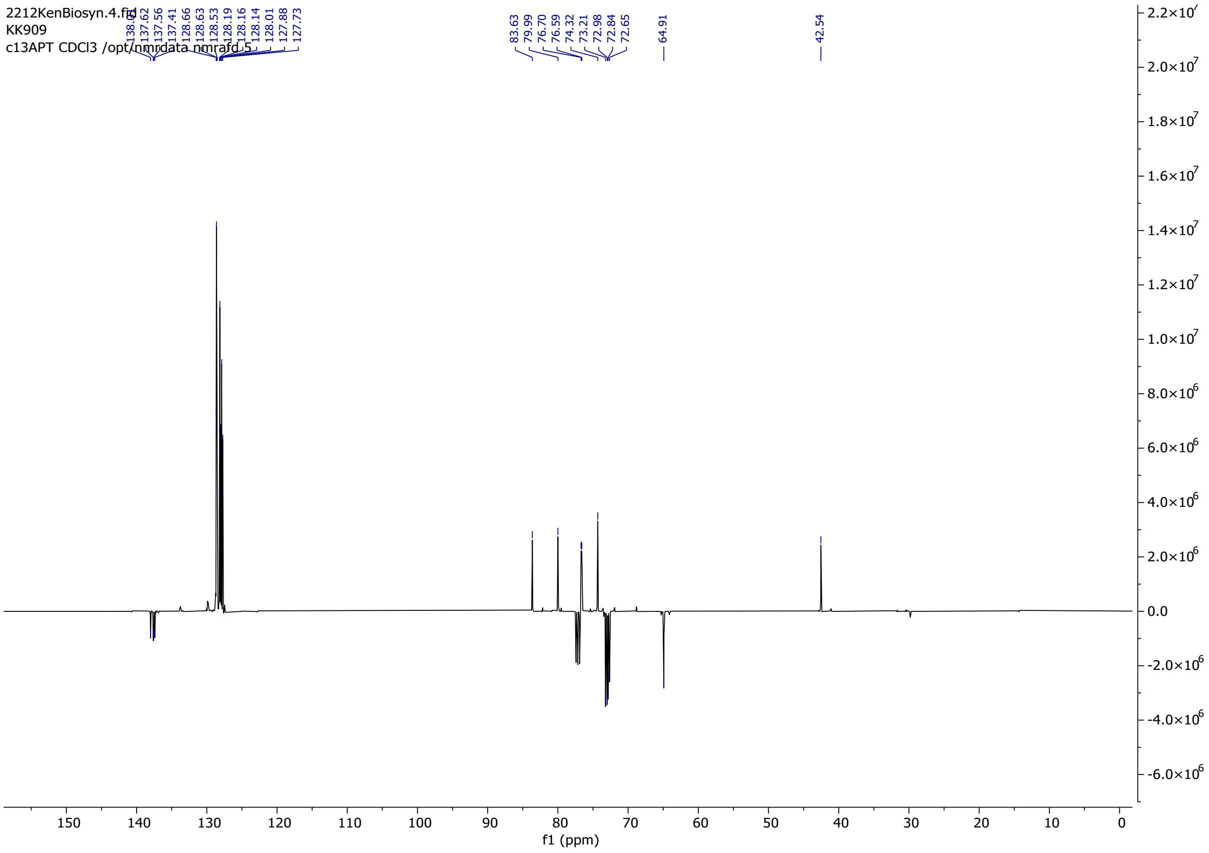

^{1}H-NMR and ^{13}C-NMR spectra of **16** in MeOD

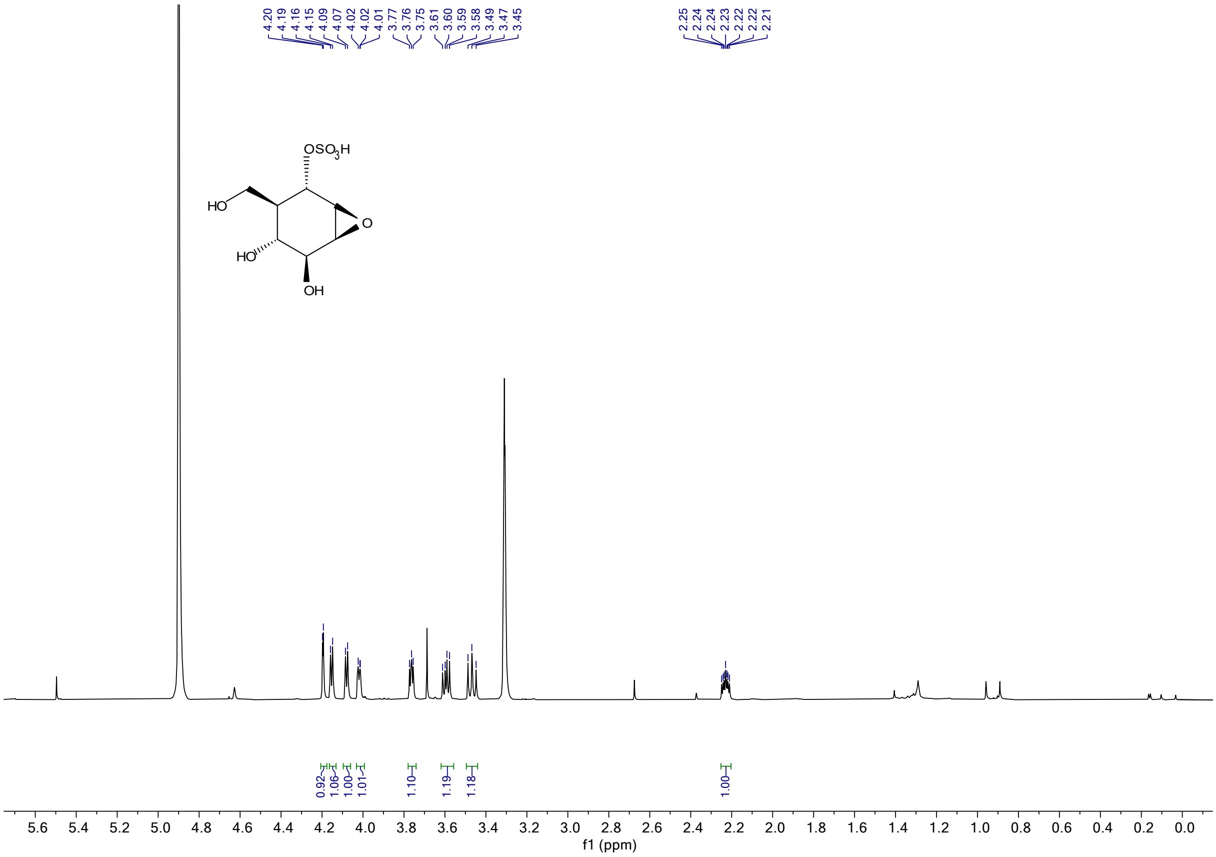

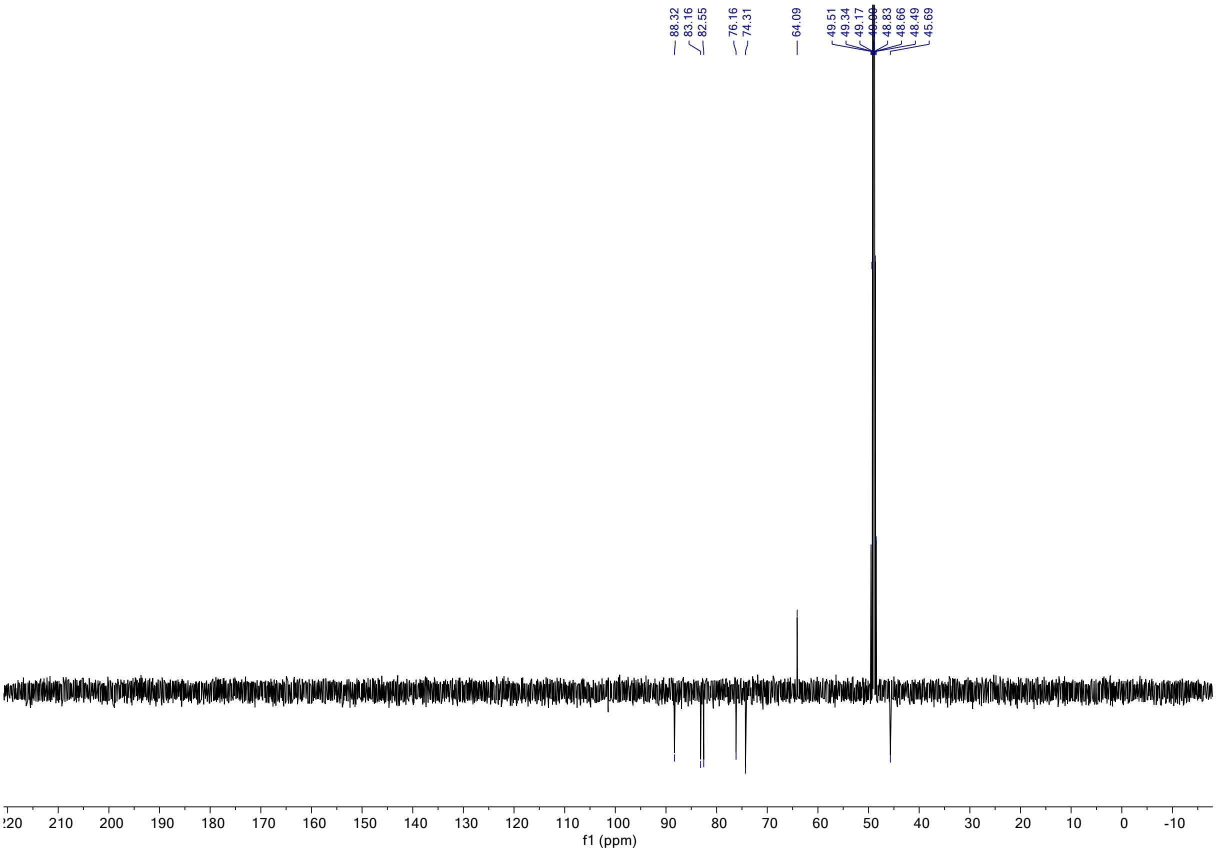

^{1}H-NMR and ^{13}C-NMR spectra of **18** in $CDCl_3$

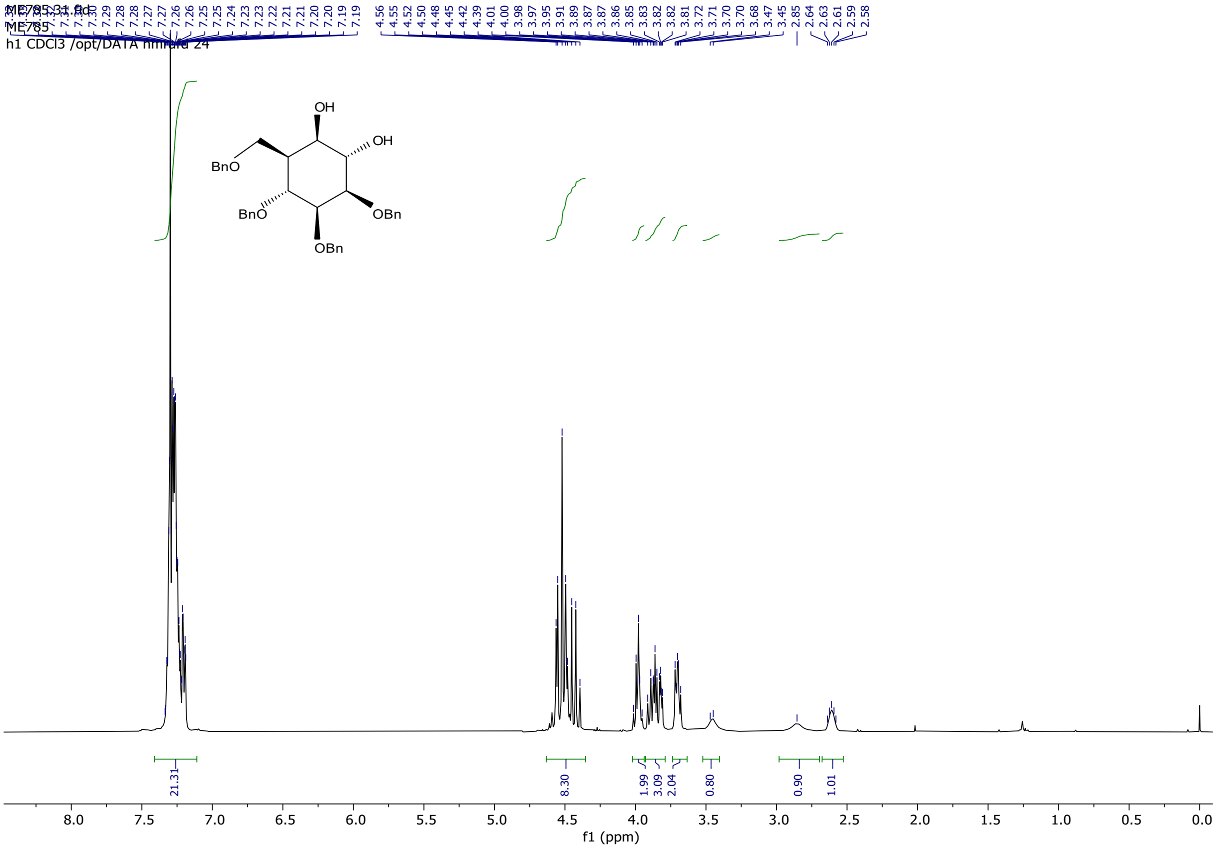

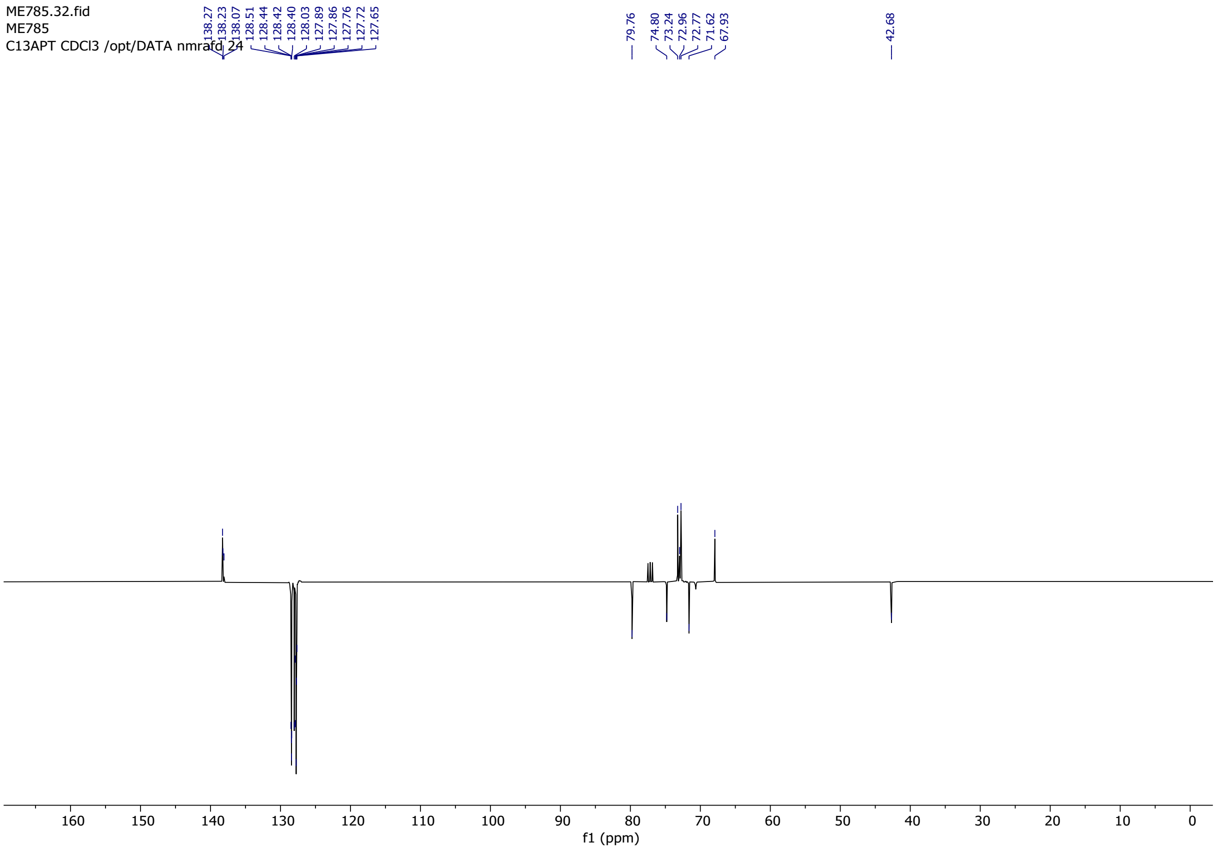

^{1}H-NMR and ^{13}C-NMR spectra of **19** in $CDCl_3$

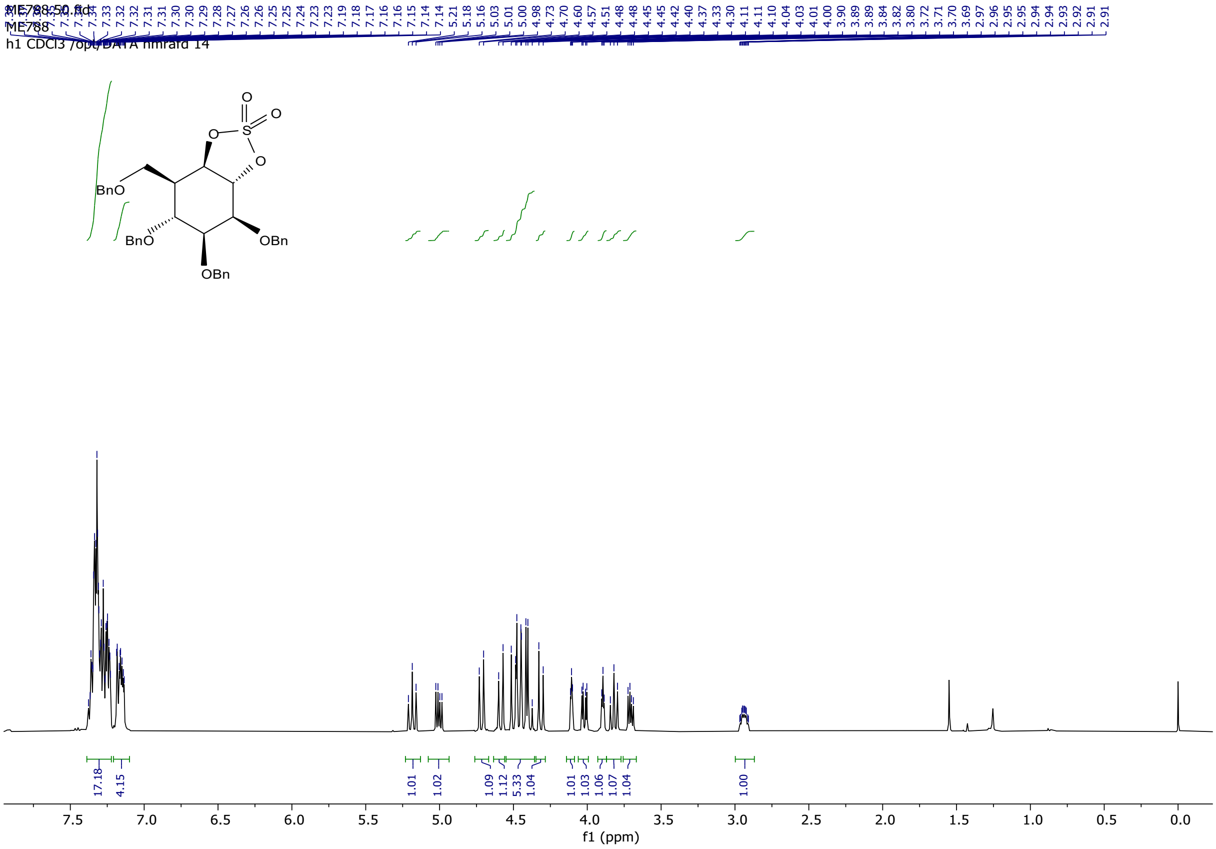

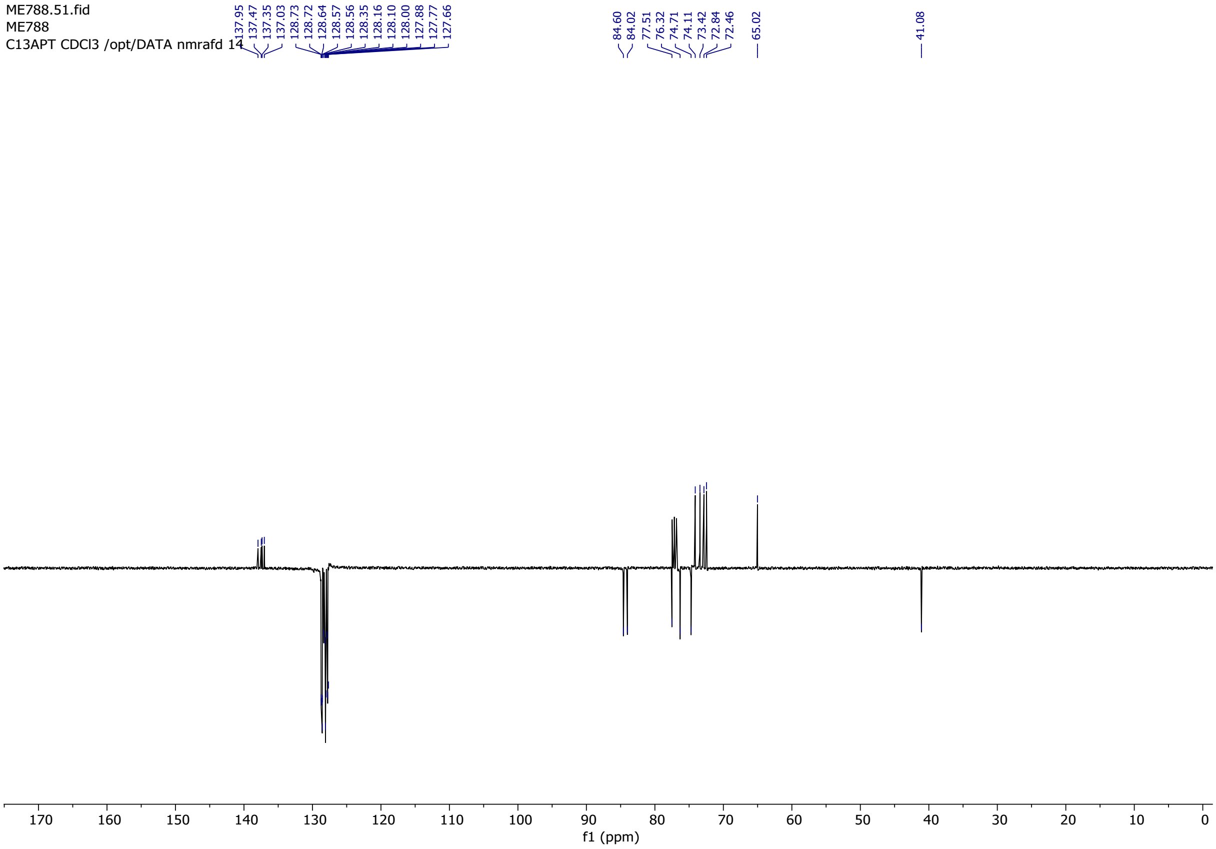

^{1}H-NMR and ^{13}C-NMR spectra of **5** in MeOD

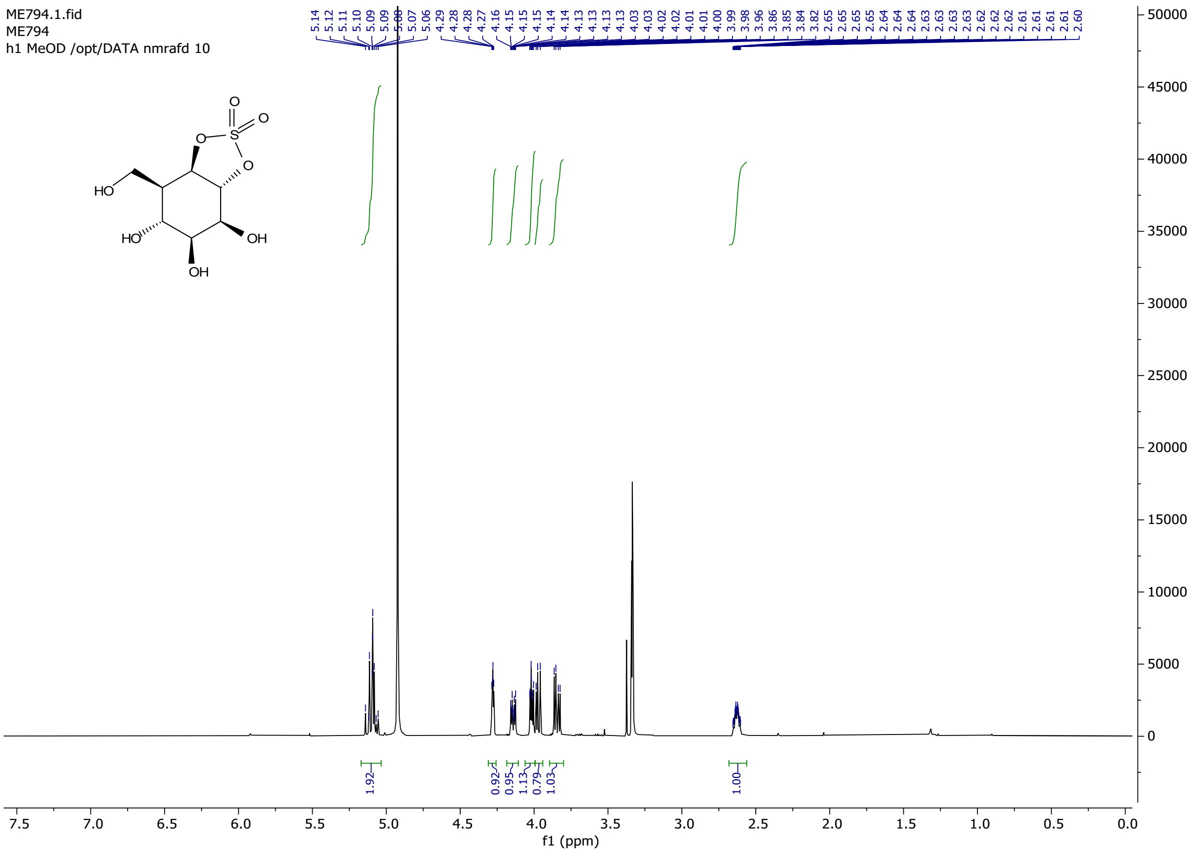

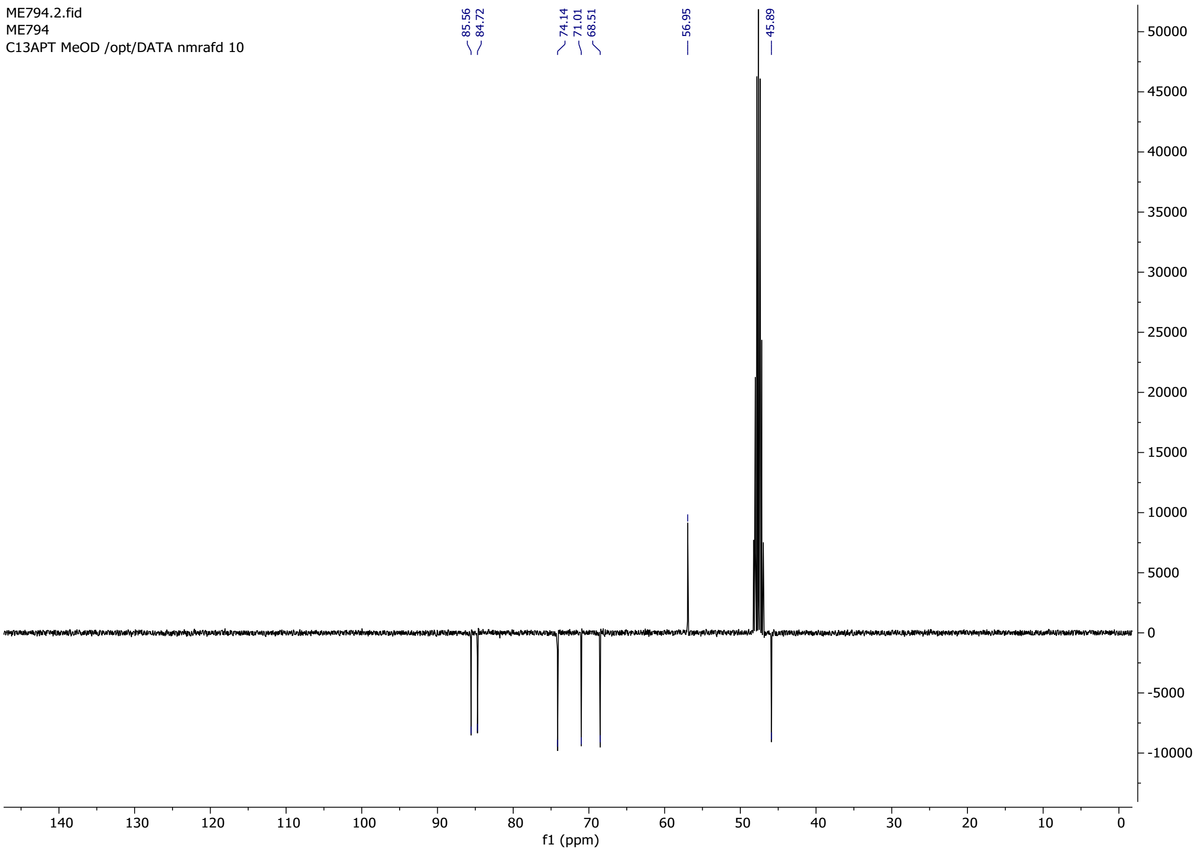

^{1}H-NMR and ^{13}C-NMR spectra of **6** in MeOD

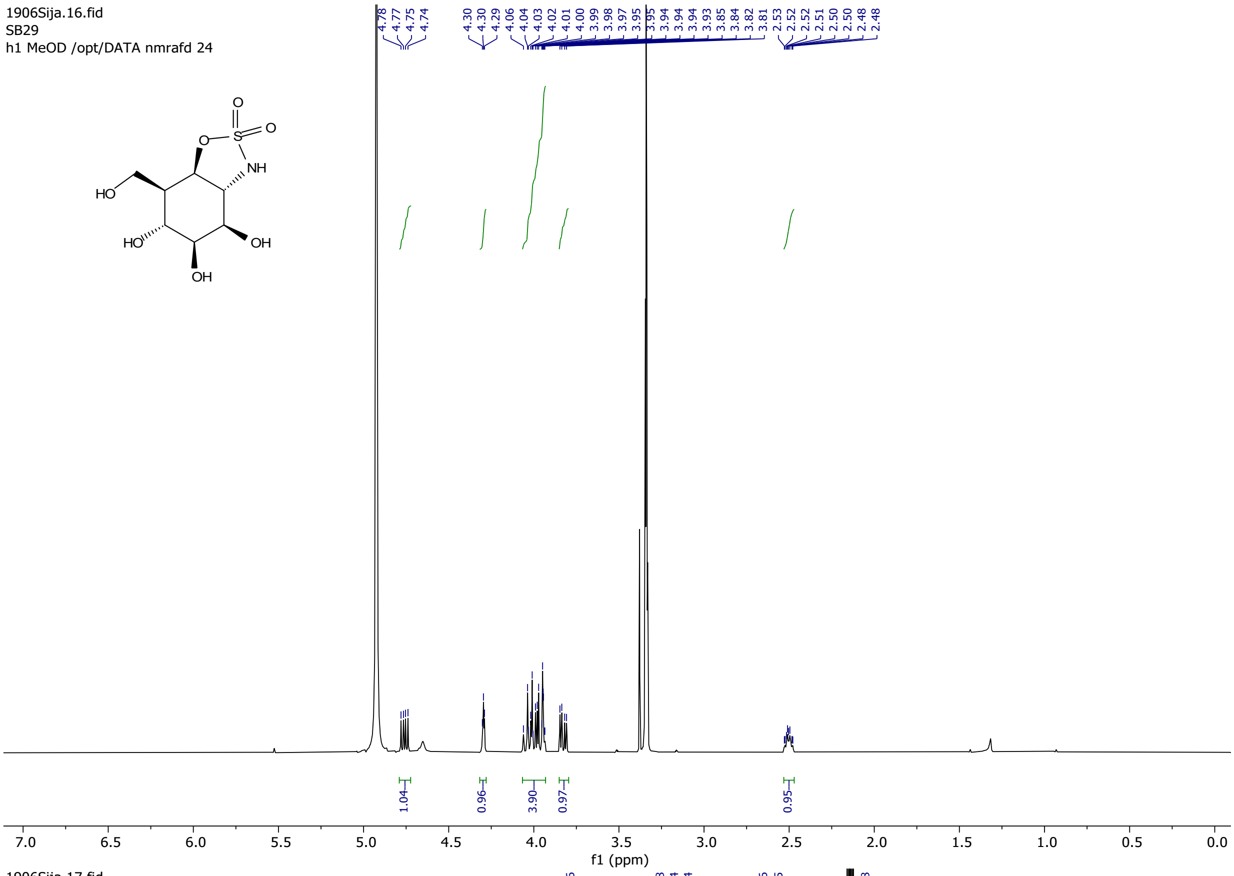
1906Sija.16.fid
SB29
h1 MeOD /opt/DATA nmrafd 24
4.78 4.77 4.75 4.74
4.30 4.30 4.29 4.06 4.04 4.03 4.02 4.01 4.00 3.99 3.98 3.97 3.95 3.95 3.94 3.94 3.94 3.93 3.85 3.84 3.82 3.81 2.53 2.52 2.52 2.51 2.50 2.50 2.48 2.48
O
O
S
O
NH
HO
HO
OH
OH
1.04
0.96
3.90
0.97
0.95
7.0 6.5 6.0 5.5 5.0 4.5 4.0 3.5 3.0 2.5 2.0 1.5 1.0 0.5 0.0
f1 (ppm)

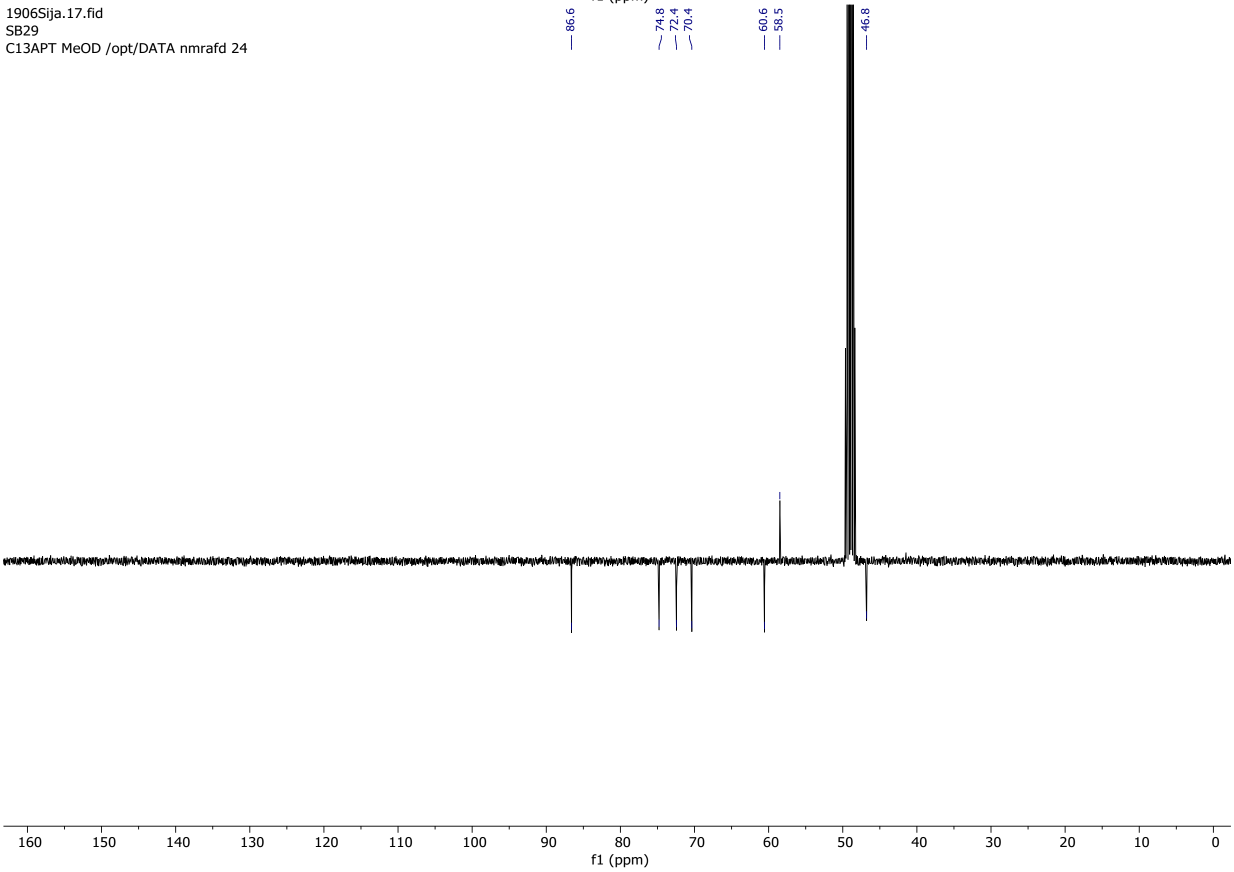
1906Sija.17.fid
SB29
C13APT MeOD /opt/DATA nmrafd 24
86.6
74.8
72.4
70.4
60.6
58.5
46.8
160 150 140 130 120 110 100 90 80 70 60 50 40 30 20 10 0
f1 (ppm)

5. References

[1] F. Vallée, K. Karaveg, A. Herscovics, K. W. Moremen, P. L. Howell, *J. Biol. Chem.* **2000**, *275*, 41287–41298.

[2] A. Males, L. Raich, S. J. Williams, C. Rovira, G. J. Davies, *ChemBioChem* **2017**, *18*, 1496–1501.

[3] A. J. Thompson, J. Dabin, J. Iglesias-Fernández, A. Ardèvol, Z. Dinev, S. J. Williams, O. Bande, A. Siriwardena, C. Moreland, T. C. Hu, et al., *Angew. Chemie - Int. Ed.* **2012**, *51*, 10997–11001.

[4] E. van Rijssel, A. Janssen, A. Males, G. Davies, G. van der Marel, H. S. Overkleeft, J. Codée, *ChemBioChem* **2017**, *18*, 1297–1304.

[5] A. W. Senior, R. Evans, J. Jumper, J. Kirkpatrick, L. Sifre, T. Green, C. Qin, A. Žídek, A. W. R. Nelson, A. Bridgland, et al., *Nature* **2020**, *577*, 706–710.

[6] F. Madeira, M. Pearce, A. R. N. Tivey, P. Basutkar, J. Lee, O. Edbali, N. Madhusoodanan, A. Kolesnikov, R. Lopez, *Nucleic Acids Res.* **2022**, *50*, W276—W279.

[7] M. D. L. Suits, Y. Zhu, E. J. Taylor, J. Walton, D. L. Zechel, H. J. Gilbert, G. J. Davies, *PLoS One* **2010**, *5*, 1–11.

[8] P. R. Evans, G. N. Murshudov, *Acta Crystallogr. Sect. D Biol. Crystallogr.* **2013**, *69*, 1204–1214.

[9] P. Emsley, B. Lohkamp, W. G. Scott, K. Cowtan, *Acta Crystallogr. D. Biol. Crystallogr.* **2010**, *66*, 486–501.

[10] G. N. Murshudov, P. Skubák, A. A. Lebedev, N. S. Pannu, R. A. Steiner, R. A. Nicholls, M. D. Winn, F. Long, A. A. Vagin, *Acta Crystallogr. Sect. D Biol. Crystallogr.* **2011**, 355–367.

[11] Z. Armstrong, C.-L. Kuo, D. Lahav, B. Liu, R. Johnson, T. J. M. Beenakker, C. de Boer, C.-S. Wong, E. R. van Rijssel, M. F. Debets, et al., *J. Am. Chem. Soc.* **2020**, *142*, 13021–13029.

[12] L. E. Tailford, V. A. Money, N. L. Smith, C. Dumon, G. J. Davies, H. J. Gilbert, *J. Biol. Chem.* **2007**, *282*, 11291—11299.
